# Visual analysis of [^18^F]FDG PET/CT imaging yields superior diagnostic accuracy compared to semiquantitative analysis in the assessment of giant cell arteritis

**DOI:** 10.1007/s00259-026-07981-5

**Published:** 2026-06-06

**Authors:** Luca Seitz, Rahel Friedli, Nasir Gözlügöl, Susana Bucher, Pascal Seitz, Fabian Lötscher, Marc Gilgen, Jennifer Amsler, Patrick Stähli, Fabrice Helfenstein, Yvonne Döring, Györgyi Hamvas, Marc Schindewolf, Axel Rominger, Britta Maurer, Lisa Christ, Federico Caobelli

**Affiliations:** 1 Department of Rheumatology and Immunology, University Hospital Bern, Inselspital, University of Bern, Bern, Switzerland; 2 Department of Nuclear Medicine, Bern University Hospital, Inselspital, University of Bern, Bern, Switzerland; 3 Graduate School for Health Sciences, University of Bern, Bern, Switzerland; 4 Department of Angiology, Inselspital, University Hospital Bern, University of Bern, Bern, Switzerland; 5 Department of Clinical Research, CTU Bern, University of Bern, Bern, Switzerland; 6 Institute for Cardiovascular Prevention (IPEK), Ludwig-Maximilians-University Munich, Munich, Germany; 7 Partner Site Munich Heart Alliance, DZHK (German Center for Cardiovascular Research), Munich, Germany; 8 Department for BioMedical Research (DBMR), University of Bern, Bern, Switzerland

**Keywords:** 18F-FDG PET/CT, Giant cell arteritis, Large vessel vasculitis, Atherosclerosis, Visual interpretation

## Abstract

**Background:**

[¹⁸F]FDG PET/CT can be used to diagnose suspected new-onset giant cell arteritis (GCA), but it is unclear, whether visual or semiquantitative evaluation yields the highest accuracy. We therefore aimed to compare both methods with regard to diagnostic accuracy within the same patient.

**Methods:**

This single centre study included all ≥ 50 year-old patients undergoing [¹⁸F]FDG PET/CT between 2012 and 2023 for suspected new-onset GCA. Scans were re-read by two nuclear medicine physicians. Thirty-three arterial segments were scored visually, either with a standard criterion (activity at least equal liver, grade ≥ 2) or with a more specific one (activity above liver, grade 3). Standardized uptake values (SUVs) were measured in each segment, also normalized to liver and blood pool. For SUVs and ratios, ROC-curves were calculated per segment, segment groups and on the patient level to determine optimal cut-offs. The reference diagnosis was determined clinically by chart review after a follow-up of ≥ 6 months.

**Results:**

A total of 163 patients, 39 (24%) with GCA, were included. Visual analysis with ≥ 1 segment with uptake grade ≥ 2 showed high sensitivity (80.0%, 95%-confidence interval (CI) 0.64–0.91) and specificity (83.0%, 95%-CI 0.75–0.89) and correct diagnosis in 82.0% (95%-CI 0.76–0.88). Using uptake grade 3, specificity was 96.0% (95%-CI 0.91–0.99) and sensitivity 59.0% (95%-CI 0.42–0.74), correct diagnosis in 87.0% (95%-CI 0.81–0.92). Semiquantitative methods proved less accurate, with SUVmax/SUVmax(liver) ratio at a threshold of 0.917 having the highest accuracy (sensitivity 87.2%, specificity 58.9%, correct diagnosis 65.6%).

**Conclusion:**

In suspected, new-onset GCA, expert visual interpretation of [¹⁸F]FDG PET/CT should be recommended, as it outperforms semiquantitative methods.

**Supplementary Information:**

The online version contains supplementary material available at https://doi.org/10.1007/s00259-026-07981-5.

## Introduction

[^18^F]-Fluorodeoxyglucose positron emission tomography/computed tomography ([^18^F]FDG-PET/CT) enables non-invasive visualization of metabolic activity in affe

cted arterial walls in large vessel vasculitis (LVV), especially giant cell arteritis (GCA) [1–3]. According to the 2023 EULAR recommendations for imaging in LVV, ultrasound is the first-line imaging modality in patients with suspected GCA, whereas [^18^F]FDG PET/CT represents an important alternative imaging modality, particularly for the assessment of extracranial large-vessel involvement [4]. Current consensus recommends a visual assessment of arterial wall uptake using a 4-point scale and based on comparison with liver uptake [1]. Grades 0 and 1 are considered normal, grade 2 is generally considered highly suggestive of vasculitis and grade 3 is interpreted as definitively pathological [1].

While visual interpretation is robust if readers are experienced, it remains subjective. Consequently, semiquantitative parameters based on standardized uptake values (SUV) have been proposed to improve reproducibility and aid diagnostic decision making [5–7]. Specifically, the ratio of arterial SUVmax to liver SUVmean (SUVmax/L-SUVmean) was recently found to be superior to other ratios or individual SUV values by using a threshold of > 1.0 for supra-aortic vessels and > 1.3 for thoracic, abdominal and pelvic vessels on analogue, conventional PET/CT systems [6, 7]. Semiquantitative values are known to vary depending on scanner type, acquisition protocol, and reconstruction algorithms [8]. This poses a challenge for generalizability of specific cut-off values, especially when digital PET/CT systems, including Long Axial Field of View (LAFOV) scanners, are used [9, 10]. Hence, while semiquantitative image analysis may complement visual interpretation, its performance in comparison to the purely visual interpretation and its generalizability to digital or even LAFOV PET/CT systems remain under investigation.

In this study, we aimed to assess the diagnostic accuracy of [^18^F]FDG PET/CT in patients with suspected GCA, also comparing the diagnostic performance of expert visual assessment with that of semiquantitative analysis. Given the increasing clinical reliance on PET imaging and the rapid evolution of imaging technologies, this information is important for optimizing diagnostic workflows and improving patient care.

## Methods

This is a retrospective and single-centre study at the University Hospital Bern, Switzerland. The study was conducted in accordance with the Declaration of Helsinki and was approved by the Ethics Committee Bern, Switzerland (ID 2023 − 00112).

### Study population

The following inclusion criteria had to be met: (1) Age ≥ 50 years; (2) [^18^F]FDG PET/CT performed between January 1st, 2012, and December 31st, 2023 with written report including a predefined set of search terms (see below); (3) available informed consent. Patients were excluded if any of the following criteria was met: (1) [^18^F]FDG PET/CT not performed for suspected new-onset LVV; (2) follow-up data or clinical information insufficient to establish an expert reference diagnosis; (3) vasculitis diagnosis other than GCA; (4) raw [^18^F]FDG PET/CT imaging data not available.

To identify eligible cases, the institutional picture archiving and communication system (PACS) was queried for [^18^F]FDG PET/CT reports containing the following search terms (translated from German): arteritis, vasculitis, temporal artery, aortitis, giant cell vasculitis, giant cell arteritis, GCA, large vessel vasculitis, Horton. All identified records were subsequently reviewed by a senior rheumatologist and vasculitis specialist (L.S. with 15 years of experience) to determine compliance with the inclusion and exclusion criteria.

### Image acquisition

Imaging was performed on 3 different scanner models: Biograph mCT (analogue PET scanner, Siemens Healthineer, Knoxville, TX, USA), Biograph Vision 600 (digital, standard-axial-field-of-view scanner, Siemens Healthineer, Knoxville, TX, USA) and Biograph Vision Quadra (digital LAFOV scanner, Siemens Healthineer, Knoxville, TX, US) for 33 (20%), 35 (21%) and 95 (58%) scans, respectively. Imaging was performed in the Clinic of Nuclear Medicine of the University Hospital Bern, Switzerland, a center certified by EARL for the harmonization across different scanners. Patients received weight-adapted [¹⁸F]FDG according to the scanner-specific institutional protocol: 3.5 MBq/kg for Siemens Biograph mCT, 3.0 MBq/kg for Siemens Vision, and 2.5 MBq/kg for Siemens Vision Quadra/LAFOV PET/CT systems. The median administered activity was 246 MBq (IQR 204–293 MBq). Glucose levels were always < 10 mmol/L (median 6.1 mmol/L). The uptake time was 60 min, 90 min and 120 min for 53 (32%), 53 (32%) and 57 (35%) patients, respectively. Because administered activity and uptake time differed according to scanner type and institutional protocol changes over time, additional subgroup analyses were performed stratified by uptake time, 60, 90, and 120 min.

Images were acquired from the skull base to mid-thigh in 14 (8.7%), from vertex to mid-thigh in 69 (42%), and from the whole body in 80 (49%) patients. Acquisition time was 3 min/bed position for Biograph mCT, 90s/bed position for Biograph Vision 600 and 10 min in a single bed position for Biograph Vision Quadra. A non-contrast-enhanced CT was performed for attenuation correction. Images were always reconstructed to a 256 × 256 matrix with an ordered-subset-expectation-maximization (OSEM) iterative algorithm, specific for each PET scanner.

### Image evaluation

[^18^F]FDG PET/CT images were re-read by two nuclear medicine physicians (FC with > 15-year experience with board certification and NG with 4-year experience) using appropriate software included in Syngo.via package (Siemens Healthineers, Knoxville, TX; US). FC re-read 65 scans (40%) and NG 98 scans (60%), respectively. For inter-reader analysis, 30 scans were re-read by both readers. Readers were blinded to all clinical information, including the original PET reporting, but were aware of age and sex of the patients. The examination date, scanner model, uptake time and [^18^F]FDG dose were available to the readers.

### Rating of arteries

In total, 33 arterial territories were evaluated per patient. Bilateral assessment was performed for the subclavian, axillary, brachial, common iliac, internal iliac, external iliac, common femoral, common carotid, internal carotid, external carotid, vertebral, superficial temporal, occipital, and facial arteries. In addition, the single midline/unpaired arterial territories, including the innominate artery, ascending aorta, aortic arch, descending thoracic aorta, and abdominal aorta, were assessed.

Large- and medium-sized arteries were initially assessed visually according to consensus recommendations using a 4-point liver-referenced scale (0, no uptake; 1, low-grade vascular uptake lower than liver; 2, intermediate vascular uptake equal to liver; and 3, high-grade vascular uptake greater than liver) [1]. A visual score ≥ 2 was considered likely pathological and a score of 3 as clearly pathological. Cranial arteries were assessed visually using background activity as reference, with uptake classified as absent, equal to background, or above background. Cranial artery uptake above background was considered pathological.

Only after completion of the visual assessment, semiquantitative SUV measurements were performed for each arterial segment. SUVmax, SUVmean, and SUVpeak were obtained by manually placing a volume of interest (VOI) with a 40% isocontour around the entire arterial diameter, while avoiding focal hot spots typical of atherosclerotic lesions. All SUV values were then automatically calculated by the software.

For calculation of SUV ratios, reference semiquantitative measurements were also obtained for the liver and the blood pool. A standard 10 cm³ VOI was placed in healthy liver tissue in the right lobe, and a standard 2 × 2-pixel ROI was positioned in the blood pool at the level of the mitral valve plane. Using these reference values, eight SUV ratios were calculated for each of the 33 arterial segments: arterial wall SUVmax to liver SUVmax, arterial wall SUVmax to liver SUVmean, arterial wall SUVmean to liver SUVmean, arterial wall SUVpeak to liver SUVpeak, arterial wall SUVmax to blood pool SUVmax, arterial wall SUVmax to blood pool SUVmean, arterial wall SUVmean to blood pool SUVmean, and arterial wall SUVpeak to blood pool SUVpeak.

### Diagnostic reference standard

The final reference diagnosis was determined by clinical chart review after a follow-up of at least 6 months after the initial diagnostic work-up. It was established by experienced vasculitis specialists (L.S., P.S. and F.L.) and was based on a comprehensive analysis of all available data in the medical records. Clinical data were extracted from individual electronic health records and entered into a coded REDCap database [11]. Blinding to [^18^F]FDG PET/CT findings in the clinical documentation was not feasible due to their integration in routine care. However, [^18^F]FDG PET/CT findings documented in the original clinical reports were not used in any analysis presented in this study.

### Statistical analysis

Statistical analyses and figures were done by a biostatistician (F.H.) with R version 4.4.1 (2024-06-14). Patient characteristics are reported as absolute and relative frequencies for categorical variables and as median with interquartile range or mean and standard deviation for continuous variables. For comparisons between groups, Fisher’s exact and the Mann-Whitney-Wilcoxon test for categorical and continuous variables, respectively, were used. The proportion of correct classifications, sensitivity, and specificity are reported as absolute and relative frequencies with 95%-confidence interval (CI), based on exact likelihood ratios. Binary agreement at the patient level was quantified by Cohen’s kappa with an analytical 95%-CI. The 95%-CI of the area under the curve (AUC) of the receiver-operating-characteristic (ROC) was estimated based on 2000 bootstraps. Best cut-offs were determined using the Youden’s formular (if it rendered more than 1 cut-off point, the larger one was chosen). Analyses were performed per-patient (all arterial segments together), per-artery (each arterial segment individually) and per-subgroup (arterial segments grouped into 7 different regions: head; neck-arm; thorax; abdomen-pelvis; neck; arm; neck-arm-thorax). The individual arterial segments in each subgroup are listed in Supplementary Table 1. For inter-rater analysis, agreement on diagnosis was determined by Cohen’s kappa and agreement on the raw SUV measurements was determined by intra-class coefficient (ICC).

## Results

### Patient cohort

From 333 patients initially identified, 170 were excluded according to predefined criteria (Supplementary Fig. 1). The final cohort comprised 163 patients with suspected new-onset GCA (50% female), of whom 39 (23.9%) received a final clinical diagnosis of GCA (Table 1). The most frequent alternative diagnoses were polymyalgia rheumatica (*n* = 48) and polyarthritis (*n* = 16). These patients were not included as a predefined control group, but represented real-world differential diagnoses among patients referred for suspected GCA in whom the final expert diagnosis did not confirm GCA.

Patients with GCA more frequently presented with cranial symptoms, new-onset headache, scalp tenderness, and abnormal temporal artery findings, and had higher inflammatory markers. The 2022 ACR/EULAR classification criteria were fulfilled in 82% of GCA patients. Cranial symptoms occurred in 20/39 (51%) and 30/124 (25%) of patients with and without GCA, respectively. Based on signs and symptoms, imaging and histology, 37/39 (95%) patients with GCA had extracranial LVV, 24/39 (62%) both cranial and large-vessel involvement, and 2/39 (5%) had cranial vasculitis only. At the time of the [^18^F]FDG PET/CT scan, 10/39 (26%) and 36/124 (29%) patients with and without GCA, respectively, were taking glucocorticoids. For all patients receiving glucocorticoids, the median dose of prednisone was 5 mg (IQR 3.75 to 15 mg, 2 missing values) and the median duration of glucocorticoid treatment was 66 days (IQR 24–200 days; 6 missing values); for the GCA patients the median dose was 35 mg (IQR 5 to 60 mg) and median duration 38 days (IQR 7-189 days; 2 missing values); for the non-GCA patients it was 5 mg (IQR 5 to 10 mg, 2 missing values) and 83 days (IQR 30 to 201 days; 4 missing values). Given the known effect of glucocorticoids on vascular [¹⁸F]FDG uptake, glucocorticoid exposure at the time of imaging was considered a potential confounder and was further evaluated in subgroup analyses. Detailed baseline characteristics are summarized in Table 1.

**Table 1:** Patients’ characteristics. ^a^, n (%) unless stated otherwise; ^b^, including (48 polymyalgia rheumatica, 16 polyarthritis, 13 fever/inflammation of unknown origin, 8 atherosclerosis, 5 collagen vascular diseases, 4 autoinflammation, 4 osteoarthritis, 4 spondyloarthritis, 3 lymphoma, 2 primary headache, 2 infection, 2 non arteriitic acute blindness, 13 other diseases); ^c^, mean (standard deviation); ^d^, median (minimum, interquartile range, maximum); ^e^, established atherosclerotic comorbidity (e.g., myocardial infarction); ^f^, persistent vision loss (complete or incomplete, unilateral or bilateral); ^g^, ≥ 1 dose of methylprednisolone (500–1000 mg) was given intravenously ≥ 1 day prior to the FDG-PET-CT; ^h^, as documented in the medical records (any imaging modality). ACR/EULAR, American College of Rheumatology/European Alliance of Associations for Rheumatology; CRP, C-reactive protein; GC, glucocorticoids; GCA, giant cell arteritis; i.v.; intravenous; n.a., not applicable; LVV, large vessel vasculitis; PET-CT; positron emission tomography - computer tomography; PETVAS, PET Vascular Activity Score; PMR, polymyalgia rheumatica; TA, temporal artery

Characteristic ^a^	Total (*N* = 163)	GCA (*N* = 39)	No GCA ^b^ (*N* = 124)	*p*-value	Missing values
Age (years) ^c^	68.9 (9.9)	70.9 (7.7)	68.3 (10.4)	0.094	0
Female patients	81 (50%)	23 (59%)	58 (47%)	0.3	0
2022 - ACR/EULAR fulfilled	n.a.	32 (82%)	n.a.	n.a.	0
2022 - ACR/EULAR (points) ^d^	4 (0, 2–6, 17)	10 (2, 6–12, 17)	4 (0, 2–4, 8)	< 0.001	0
Atherosclerosis ^e^	45 (28%)	7 (18%)	38 (31%)	0.3	1
Constitutional symptoms	75 (46%)	19 (49%)	56 (46%)	> 0.9	4
Any cranial symptoms	50 (31%)	20 (51%)	30 (24%)	0.004	3
New-onset headache	35 (21%)	17 (44%)	18 (15%)	< 0.001	7
Scalp tenderness	16 (10%)	10 (26%)	6 (5%)	< 0.001	12
Jaw claudication	10 (6%)	4 (10%)	6 (5%)	0.4	12
Diplopia	7 (4%)	3 (8%)	4 (3%)	0.022	10
Vision loss ^f^	5 (3%)	0 (0%)	5 (4%)	0.2	13
PMR symptoms	101 (62%)	28 (72%)	73 (59%)	0.3	3
Abnormal temporal artery exam	5 (3%)	4 (10%)	1 (1%)	0.008	21
CRP (mg/L) ^d^	55 (0, 13–121, 460)	96 (2, 35–147, 460)	51 (0, 12–105, 342)	0.051	10
GC installed at time of PET-CT	46 (28%)	10 (26%)	36 (29%)	> 0.9	10
GC i.v. before PET-CT ^g^	5 (3%)	1 (3%)	4 (3%)	> 0.9	0
TA histology with vasculitis	6/27 (22%)	6/8 (75%)	0/19 (0%)	0.007	0
Imaging with LVV ^h^	n.a.	37 (95%)	n.a.	n.a.	0
Imaging or histology with cranial vasculitis ^h^	n.a.	23 (59%)	n.a.	n.a.	0
PETVAS ^d^	0 (0, 0–6, 27)	8 (0, 0–22, 27)	0 (0, 0–3, 18)	< 0.001	0

### Diagnostic performance of visual FDG-PET/CT interpretation

Visual assessment demonstrated high diagnostic accuracy for GCA at the patient level (Table 2). Using the presence of at least one arterial segment with uptake ≥ grade 2 as the diagnostic criterion resulted in high sensitivity (0.80) and good specificity (0.83), yielding an overall accuracy of 0.82 and a low negative LR, indicating good rule-out capability. A total of 30/39 (77%) patients with GCA and 15/124 (12%) without GCA were rated as vasculitis.

**Table 2:** Measures of diagnostic accuracy for visual analysis compared to the reference diagnosis. ^a^, proportion (95%-confidence interval); ^b^, ratio (95%-confidence interval). GCA, giant cell arteritis; NPV, negative predictive value; PPV, positive predictive value; LR, likelihood ratio; n.a., not applicable

	Sensitivity ^a^	Specificity ^a^	Accuracy ^a^	PPV ^a^	NPV ^a^	Positive LR ^b^	Negative LR ^b^
Total study population (*N* = 163, GCA *N* = 39)							
Visual (≥ 1 artery grade 3)	0.59 (0.42–0.74)	0.96 (0.91–0.99)	0.87 (0.81–0.92)	0.82 (0.63–0.94)	0.88 (0.82–0.93)	14.63 (5.96–35.89)	0.43 (0.29–0.62)
Visual (≥ 1 artery grade 2 or 3)	0.80 (0.64–0.91)	0.83 (0.75–0.89)	0.82 (0.76–0.88)	0.60 (0.45–0.73)	0.93 (0.86–0.97)	4.69 (3.08–7.15)	0.25 (0.13–0.46)
Glucocorticoids at the time of FDG-PET-CT (*N* = 46; GCA *N* = 10)							
Visual (≥ 1 artery grade 3)	0.70 (0.35–0.93)	0.94 (0.81–0.99)	0.89 (0.76–0.96)	0.78 (0.40–0.97)	0.92 (0.78–0.98)	12.6 (3.09–51.44)	0.32 (0.12–0.82)
Visual (≥ 1 artery grade 2 or 3)	0.90 (0.56-1.00)	0.78 (0.61–0.90)	0.80 (0.66–0.91)	0.53 (0.28–0.77)	0.97 (0.82-1.00)	4.05 (2.13–7.72)	0.13 (0.02–0.83)
No glucocorticoids at the time of FDG-PET-CT (*N* = 107; GCA *N* = 24)							
Visual (≥ 1 artery grade 3)	0.58 (0.37–0.78)	0.96 (0.90–0.99)	0.88 (0.80–0.93)	0.82 (0.57–0.96)	0.89 (0.81–0.95)	16.14 (5.05–51.55)	0.43 (0.27–0.70)
Visual (≥ 1 artery grade 2 or 3)	0.79 (0.58–0.93)	0.86 (0.76–0.92)	0.84 (0.76–0.91)	0.61 (0.42–0.78)	0.94 (0.86–0.98)	5.48 (3.12–9.61)	0.24 (0.11–0.53)
Imaging performed on Biograph or Vision (*N* = 68; GCA *N* = 21)							
Visual (≥ 1 artery grade 3)	0.52 (0.30–0.74)	1 (0.96-1.00)	0.85 (0.75–0.93)	1 (0.72-1.00)	0.83 (0.70–0.91)	n.a.	0.48 (0.30–0.75)
Visual (≥ 1 artery grade 2 or 3)	0.71 (0.48–0.89)	0.81 (0.67–0.91)	0.78 (0.66–0.87)	0.63 (0.41–0.81)	0.86 (0.73–0.95)	3.73 (1.95–7.12)	0.35 (0.18–0.71)
Imaging performed on Vision Quadra (*N* = 95, GCA *N* = 18)							
Visual (≥ 1 artery grade 3)	0.67 (0.41–0.87)	0.94 (0.86–0.98)	0.88 (0.80–0.94)	0.71 (0.44–0.90)	0.92 (0.84–0.97)	10.27 (4.14–25.46)	0.36 (0.19–0.69)
Visual (≥ 1 artery grade 2 or 3)	0.89 (0.65–0.99)	0.84 (0.74–0.92)	0.85 (0.77–0.92)	0.57 (0.37–0.76)	0.97 (0.90-1.00)	5.70 (3.31–9.84)	0.13 (0.04–0.49)

Applying a stricter visual threshold (≥ 1 arterial segment with grade 3 uptake) substantially increased specificity (0.96) and positive likelihood ratio, with an overall accuracy of 0.87, at the cost of reduced sensitivity (0.59). A total of 23/39 (59%) patients with GCA and 5/124 (4%) without GCA were rated as vasculitis. Thus, visual grade 2 optimized sensitivity, whereas grade 3 maximized specificity and rule-in performance.

Subgroup analyses showed comparable diagnostic accuracy in patients with and without glucocorticoid treatment at the time of imaging, with consistent sensitivity–specificity trade-offs across thresholds (Table 2), although confidence intervals were wide because of the small subgroup sizes. Scanner-specific analyses likewise demonstrated similar overall performance across analogue, digital, and LAFOV PET/CT systems, with overlapping confidence intervals and no clinically relevant differences in accuracy.

### Performance of semiquantitative analysis using published cut-offs

Reference SUVmax, SUVmean and SUVpeak for the liver and blood pool are shown in Supplementary Table 2. for the total population and GCA and non-GCA patients. Values were lower in GCA patients compared to non-GCA patients. Two regional grouping strategies were evaluated. Version 1 used an exploratory grouping with Region 1 including head-neck-arm-thorax and Region 2 including abdomen-pelvis. Version 2 followed the previously published supra-aortic versus aorto-iliofemoral grouping, with Region 1 including head-neck-arm vessels and Region 2 including thorax-abdomen-pelvis vessels.

When applying previously published SUVmax/SUVmean(liver) cut-offs according to the previously published regional grouping (Version 2, Table 3), semiquantitative analysis yielded very high sensitivity (0.95–1.00) and negative predictive value, but consistently poor specificity (0.17–0.26), resulting in low overal diagnostic accuracy (~ 0.40). Positive LR were close to 1, indicating minimal rule-in value. These findings were consistent across scanner types.

**Table 3:** Measures of diagnostic accuracy using previously published cut-offs for the SUVmax/SUVmeanliver ratio compared to the reference diagnosis. Cut-off > 1.3 was used for “Region 2” for all scanner types. For “Region 1” for Biograph and Vision scanners the cut-off was > 1.0 and for Vision Quadra it was > 0.8. CI, confidence interval; LR, likelihood ratio; NPV, negative predictive value; n.a., not applicable; PPV, positive predictive value

Scanner type	*N*	Prevalence	Sensitivity	Specificity	Accuracy	PPV	NPV	Positive LR	Negative LR
Version 1: Region 1 (head-neck-arm-thorax) and Region 2 (abdomen-pelvis)									
Irrespective of scanner type, but with scanner-specific thresholds	163	0.239 (0.176–0.312)	0.974 (0.865–0.999)	0.226 (0.156–0.310)	0.405 (0.329–0.484)	0.284 (0.209–0.368)	0.966 (0.822–0.999)	1.259 (1.130–1.402)	0.114 (0.016–0.808)
Only Biograph/Vision	68	0.309 (0.202–0.433)	0.952 (0.762–0.999)	0.170 (0.076–0.308)	0.412 (0.294–0.538)	0.339 (0.221–0.474)	0.889 (0.518–0.997)	1.148 (0.977–1.348)	0.280 (0.037–2.097)
Only Vision Quadra	95	0.189 (0.116–0.283)	1.000 (0.815-1.000)	0.260 (0.166–0.372)	0.400 (0.301–0.506)	0.240 (0.149–0.353)	1.000 (0.832-1.000)	1.351 (1.183–1.542)	n.a.
Version 2: Region 1 (head-neck-arm) and Region 2 (thorax-abdomen-pelvis)									
Irrespective of scanner type, but with scanner-specific thresholds	163	0.239 (0.176–0.312)	0.949 (0.827–0.994)	0.161 (0.101–0.238)	0.350 (0.277–0.428)	0.262 (0.192–0.343)	0.909 (0.708–0.989)	1.131 (1.017–1.258)	0.318 (0.078-1.300)
Only Biograph/Vision	68	0.309 (0.202–0.433)	0.905 (0.696–0.988)	0.319 (0.191–0.471)	0.500 (0.376–0.624)	0.373 (0.241–0.519)	0.882 (0.636–0.985)	1.329 (1.045–1.689)	0.298 (0.075–1.190)
Only Vision Quadra	95	0.189 (0.116–0.283)	1.000 (0.815-1.000)	0.065 (0.021–0.145)	0.242 (0.160–0.341)	0.200 (0.123–0.298)	1.000 (0.478-1.000)	1.069 (1.008–1.134)	n.a.

Overall, published semiquantitative cut-offs effectively excluded GCA, but provided limited diagnostic discrimination and substantially inferior accuracy compared with visual interpretation.

### ROC-derived semiquantitative cut-offs

ROC analyses were performed for multiple SUV-based parameters at the patient level, artery subgroup level, and individual artery level (Tables 4 and 5 and Supplementary Tables 3–7). Applied on all arteries combined, artery SUVmax normalized to liver SUVmax achieved the highest overall accuracy (0.66), with high sensitivity (0.87) but only moderate specificity (0.59). Other SUV ratios showed similar sensitivity but lower specificity.

**Table 4:** Measures of diagnostic accuracy using cut-offs derived from ROC at patient level - (all arteries, all 163 patients): CI, confidence interval; LR, likelihood ratio; NPV, negative predictive value; PPV, positive predictive value; SUV, standardized uptake value

Parameter	Threshold (Youden)	Sensitivity	Specificity	Accuracy	PPV	NPV	Positive LR	Negative LR
Artery SUVmax	2.525	0.897 (0.758–0.971)	0.403 (0.316–0.495)	0.521 (0.442-0.600)	0.321 (0.235–0.417)	0.926 (0.821–0.979)	1.504 (1.257–1.799)	0.254 (0.098–0.660)
Artery SUVmean	1.975	0.821 (0.665–0.925)	0.476 (0.385–0.567)	0.558 (0.479–0.636)	0.330 (0.238–0.433)	0.894 (0.794–0.956)	1.565 (1.253–1.956)	0.377 (0.188–0.757)
Artery SUVpeak	2.225	0.821 (0.665–0.925)	0.573 (0.481–0.661)	0.632 (0.553–0.706)	0.376 (0.274–0.488)	0.910 (0.824–0.963)	1.920 (1.493–2.468)	0.313 (0.158–0.624)
Artery SUVmax/liver SUVmax	0.917	0.872 (0.726–0.957)	0.589 (0.497–0.676)	0.656 (0.578–0.729)	0.400 (0.295–0.512)	0.936 (0.857–0.979)	2.120 (1.663–2.701)	0.218 (0.095-0.500)
Artery SUVmax/liver SUVmean	1.081	0.974 (0.865–0.999)	0.290 (0.212–0.379)	0.454 (0.376–0.534)	0.302 (0.223–0.390)	0.973 (0.858–0.999)	1.373 (1.213–1.554)	0.088 (0.013–0.623)
Artery SUVmean/liver SUVmean	0.862	0.923 (0.791–0.984)	0.371 (0.286–0.462)	0.503 (0.424–0.582)	0.316 (0.232–0.409)	0.939 (0.831–0.987)	1.467 (1.247–1.727)	0.207 (0.068–0.630)
Artery SUVpeak/liver SUVpeak	0.801	0.897 (0.758–0.971)	0.371 (0.286–0.462)	0.497 (0.418–0.576)	0.310 (0.226–0.404)	0.920 (0.808–0.978)	1.427 (1.201–1.694)	0.276 (0.106–0.719)
Artery SUVmax/blood SUVmax	1.229	0.949 (0.827–0.994)	0.484 (0.393–0.575)	0.595 (0.516–0.671)	0.366 (0.273–0.468)	0.968 (0.888–0.996)	1.838 (1.527–2.213)	0.106 (0.027–0.414)
Artery SUVmax/blood SUVmean	1.541	0.949 (0.827–0.994)	0.387 (0.301–0.479)	0.521 (0.442-0.600)	0.327 (0.242–0.422)	0.960 (0.863–0.995)	1.548 (1.322–1.812)	0.132 (0.034–0.520)
Artery SUVmean/blood SUVmean	1.175	0.974 (0.865–0.999)	0.403 (0.316–0.495)	0.540 (0.460–0.618)	0.339 (0.253–0.435)	0.980 (0.896-1.000)	1.633 (1.401–1.903)	0.064 (0.009–0.445)
Artery SUVpeak/blood SUVpeak	1.070	0.923 (0.791–0.984)	0.355 (0.271–0.446)	0.491 (0.412–0.570)	0.310 (0.228–0.403)	0.936 (0.825–0.987)	1.431 (1.221–1.677)	0.217 (0.071–0.660)

**Table 5:** Measures of diagnostic accuracy using cut-offs derived from ROC at patient level - (all arteries except cranial vessels, all 163 patients): CI, confidence interval; LR, likelihood ratio; NPV, negative predictive value; PPV, positive predictive value; SUV, standardized uptake value

Parameter	Threshold (Youden)	Sensitivity	Specificity	Accuracy	PPV	NPV	Positive LR	Negative LR
Artery SUVmax	2.525	0.897 (0.758–0.971)	0.403 (0.316–0.495)	0.521 (0.442-0.600)	0.321 (0.235–0.417)	0.926 (0.821–0.979)	1.504 (1.257–1.799)	0.254 (0.098–0.660)
Artery SUVmean	1.975	0.795 (0.635–0.907)	0.484 (0.393–0.575)	0.558 (0.479–0.636)	0.326 (0.234–0.430)	0.882 (0.781–0.948)	1.540 (1.220–1.945)	0.424 (0.223–0.807)
Artery SUVpeak	2.225	0.795 (0.635–0.907)	0.573 (0.481–0.661)	0.626 (0.547-0.700)	0.369 (0.266–0.481)	0.899 (0.810–0.955)	1.860 (1.436–2.409)	0.358 (0.190–0.677)
Artery SUVmax/liver SUVmax	0.808	0.923 (0.791–0.984)	0.355 (0.271–0.446)	0.491 (0.412–0.570)	0.310 (0.228–0.403)	0.936 (0.825–0.987)	1.431 (1.221–1.677)	0.217 (0.071–0.660)
Artery SUVmax/liver SUVmean	1.113	0.974 (0.865–0.999)	0.347 (0.264–0.437)	0.497 (0.418–0.576)	0.319 (0.237–0.411)	0.977 (0.880–0.999)	1.492 (1.299–1.712)	0.074 (0.011–0.520)
Artery SUVmean/liver SUVmean	0.862	0.897 (0.758–0.971)	0.379 (0.293–0.471)	0.503 (0.424–0.582)	0.312 (0.228–0.407)	0.922 (0.811–0.978)	1.445 (1.215–1.719)	0.271 (0.104–0.703)
Artery SUVpeak/liver SUVpeak	0.801	0.872 (0.726–0.957)	0.371 (0.286–0.462)	0.491 (0.412–0.570)	0.304 (0.220–0.398)	0.902 (0.786–0.967)	1.386 (1.156–1.661)	0.346 (0.148–0.808)
Artery SUVmax/blood SUVmax	1.229	0.949 (0.827–0.994)	0.484 (0.393–0.575)	0.595 (0.516–0.671)	0.366 (0.273–0.468)	0.968 (0.888–0.996)	1.838 (1.527–2.213)	0.106 (0.027–0.414)
Artery SUVmax/blood SUVmean	1.441	0.974 (0.865–0.999)	0.242 (0.170–0.327)	0.417 (0.341–0.497)	0.288 (0.212–0.373)	0.968 (0.833–0.999)	1.285 (1.149–1.437)	0.106 (0.015–0.752)
Artery SUVmean/blood SUVmean	1.175	0.974 (0.865–0.999)	0.403 (0.316–0.495)	0.540 (0.460–0.618)	0.339 (0.253–0.435)	0.980 (0.896-1.000)	1.633 (1.401–1.903)	0.064 (0.009–0.445)
Artery SUVpeak/blood SUVpeak	1.070	0.897 (0.758–0.971)	0.363 (0.278–0.454)	0.491 (0.412–0.570)	0.307 (0.224-0.400)	0.918 (0.804–0.977)	1.409 (1.188–1.670)	0.283 (0.109–0.736)

Excluding cranial arteries slightly reduced diagnostic performance for most parameters, although SUVmax normalized to liver-based ratios retained high sensitivity and low negative likelihood ratios (Table 5).

Subgroup analyses demonstrated marked heterogeneity in the parameters yielding maximal accuracy depending on artery subgroup (Supplementary Table 3). To-liver-normalized SUV ratios performed best in head and abdominal–pelvic regions, whereas absolute SUVpeak performed best in the neck–arm–thorax region. Importantly, ROC curves typically showed a smooth, gradual increase in sensitivity without a distinct inflection point (Supplementary Fig. 2), indicating the absence of robust, well-defined cut-off values.

### Subgroup analysis based on uptake time

In subgroup analyses stratified by uptake time, visual diagnostic accuracy remained broadly comparable across the 60-, 90-, and 120-minute subgroups, although confidence intervals were wide because of the smaller subgroup sizes (Supplementary Table 8). Similarly, application of previously published SUVmax/SUVmean(liver) cut-offs within each uptake-time subgroup, using scanner-specific thresholds, showed consistently high sensitivity but poor specificity and low overall accuracy, without evidence of improved diagnostic discrimination at any uptake time (Supplementary Table 9). ROC-derived semiquantitative cut-offs calculated separately for each uptake-time subgroup also showed no consistent improvement compared with the analysis of the overall cohort (Supplementary Table 10).

### Inter-rater analysis

Inter-rater analysis in a subset of 30 patients showed almost identical performance for visual analysis (grade 2 or 3) with a sensitivity of 0.80 and 0.73, specificity of 0.87 and 1.00 and accuracy of 0.83 and 0.87 for reader 1 and reader 2, respectively. Agreement was substantial with a Cohen’s kappa of 0.80 (95%-CI 0.57–1.00). Accuracy using published cut-offs for SUVmax/SUVmean(liver) was again almost identical with a sensitivity of 0.93 and 0.93, specificity of 0.13 and 0.20 and accuracy of 0.53 and 0.57 for reader 1 and reader 2, respectively. Again, agreement was substantial with a Cohen’s kappa of 0.84 (95%-CI 0.52–1.00). For the measurement of the SUV values, an almost perfect agreement for SUVmax, SUVmean and SUVpeak in 739 segments was observed with an intra-class coefficient (ICC) of 0.881 (95%- CI 0.864–0.897), 0.871 (95%- CI 0.853–0.888) and 0.884 (95%- CI 0.866–0.898), respectively. Bland-Altman plots for SUV values showed a very low systematic bias between readers. Lower and upper limits of agreements were approximately +/- 0.7–0.8 for SUVmean and SUVpeak and +/- 1 for SUVmax. Increased variability and occasional outliers at higher SUV values were observed (Supplementary Figs. 3–4).

## Discussion

This study compared the diagnostic accuracy of [¹⁸F]FDG PET/CT using visual or semiquantitative analysis in a large, real-world cohort of 163 patients with suspected GCA. The main finding is that visual assessment yields higher diagnostic accuracy than semiquantitative evaluation.

Measures of diagnostic accuracy for visual analysis in our study (sensitivity 0.80, specificity 0.83) were comparable to pooled values according to the recent systematic review informing the 2023 EULAR recommendations on imaging of LVV (sensitivity 80%, specificity 91%) [12]. As expected, sensitivity dropped (59%) and specificity rose (96%) if the ≥ 1 artery grade 3 threshold was used for diagnosis.

Conversely, semiquantitative evaluation was clearly outperformed by visual interpretation and this finding requires a detailed discussion. To date, there is no consensus on which SUV or SUV ratio and corresponding cut-off should be used, and a high heterogeneity of “best thresholds” exists in the literature, thus hampering comparisons among studies [13]. Moreover, several studies have assessed the diagnostic performance of [¹⁸F]FDG PET/CT in GCA comparing visual with semiquantitative analysis, most using predefined control groups rather than patients suspected of having GCA [1]. Lehmann et al. assessed 20 patients with LVV and 20 oncological controls and reported a sensitivity of 65% and specificity of 80% for visual interpretation, whereas arterial SUVmax (cut-off 1.78) showed higher sensitivity (90%) but low specificity (45%) [14]. Stellingwerff et al. performed a similar comparison in 18 GCA patients (12 without and 6 with glucocorticoids) and 53 controls, demonstrating a slightly higher sensitivity for visual analysis (83%) compared with aorta SUVmax/SUVmax(liver) (72%), with comparable specificity (91% vs. 92%) at a cut-off of 1.03 [15]. Nienhuis et al. assessed only cranial arteries in 24 GCA patients and 24 oncological controls, reporting sensitivities of 83% versus 79% and specificities of 75% versus 92% for visual assessment and SUVmax (cut-off 5.0), respectively, with only moderate inter-method agreement [5]. Similarly focusing on cranial arteries, Rottenburger et al. studied 34 patients suspected of GCA (17 with GCA) and found identical sensitivity (53%) and specificity (100%) for visual analysis and SUVmax/SUVmean(liver) at a cut-off of 1.0 [16]. Other studies without direct comparison to visual analysis commonly identified artery SUVmax/SUVmax(liver) or artery SUVmax/SUVmean(liver) ratios as the most accurate parameters, with cut-offs between 1.0 and 1.3 and typically higher values for abdominal and iliofemoral arteries [6, 7, 13, 17]. With regard to the difficulty in obtaining a highly accurate semiquantitative parameter with a clear cut-off, our study is no exception.

But our study is unique in that it represents by far the largest cohort to date, it includes only patients with suspected GCA (i.e. no patients with confirmed GCA prior to PET imaging), and directly compares visual assessment with semiquantitative methods within the same patients. Moreover, unlike previous reports, our semiquantitative evaluation was conducted at 3 hierarchical levels (patient, arterial groups, arterial segments) and included SUVmax, SUVmean, and SUVpeak, as well as 8 derived normalization ratios (4 liver-referenced and 4 blood-pool–referenced), all tested using ROC curve analysis. Our analysis showed no clearly defined optimal cut-off, as no single threshold provides a clinically acceptable balance between sensitivity and specificity. Instead, multiple neighboring thresholds yield comparable diagnostic performance, resulting in a relatively broad and uncertain cut-off region. Such a pattern suggests limited robustness of a single threshold and implies that dichotomization based on one cut-off should be interpreted cautiously in the setting of clinical routine.

In fact, although artery SUVmax/SUVmax(liver) achieved the highest numerical accuracy at the patient level in our cohort, still the values prevent to recommend this parameter as the preferred in suspected GCA for general use, wherein visual interpretation carried out by expert readers should be recommended. It should also be noted, that the recently published cut-offs for the artery SUVmax/SUVmean(liver) ratio [6, 7, 10] resulted in very low diagnostic accuracy (approximately 0.4) in our patients’ cohort, driven by poor specificity. The interchangeability of such thresholds is only expected to be reliable across different institutions if factors influencing their quantification, such as voxel size, image filtering, and scanner characteristics, are standardized across platforms, which is hardly the case in clinical practice. This may also explain why semiquantitative analysis proved less accurate than visual interpretation in our study, although we should consider that visual interpretation was not affected by different scanners type, hence providing evidence that in a real-world situation, wherein scanners and protocols are diverse, visual interpretation may represent the ideal choice.

This holds true even after trying to group different vessels according to their tendency to be affected by atherosclerotic alterations. As atherosclerosis is most prominent in the abdominal and pelvic region, we grouped the thoracic aorta together with the head, arm, and neck arteries in order to apply different cut-off values which may account for confounding lesions. However, accuracy remained suboptimal both with this new grouping and with that originally suggested by Imfeld et al. [6].

The lower SUVmax, SUVmean, and SUVpeak values in the liver and blood pool observed in patients with GCA were unexpected. Glucocorticoid therapy is known to increase hepatic FDG uptake, and although the average glucocorticoid dose was significantly higher in GCA patients than in non-GCA patients, treatment duration was clearly shorter in the GCA group, which may have been the more relevant factor in this population [15, 18]. Explaining the lower blood pool SUVs is more challenging, as glucocorticoids are generally not known to influence these values. While the administered FDG dose was higher in non-GCA patients, this likely reflects the higher proportion of male patients in this group (53% vs. 41%). Age was nearly identical between groups and therefore cannot account for the observed differences.

Glucocorticoid treatment at the time of PET acquisition is an important consideration, as treatment may reduce vascular [¹⁸F]FDG uptake and thereby affect both visual and semiquantitative assessment, although previous data suggest that short-term high-dose glucocorticoid treatment attenuates vascular uptake with limited impact on diagnostic accuracy [19].

In routine clinical practice, [¹⁸F]FDG PET/CT is frequently performed after glucocorticoid therapy has already been initiated. Therefore, while glucocorticoid exposure is an important potential confounder, restricting diagnostic accuracy studies exclusively to treatment-naïve patients or to patients with very short treatment intervals would limit applicability to real-world diagnostic pathways. In the present cohort, 28% of patients were receiving glucocorticoids at the time of imaging, which reflects this real-world setting. Importantly, all patients were referred for suspected new-onset GCA in the context of clinically active disease, and the primary aim was to compare visual and semiquantitative assessment within the same scans. Thus, any treatment-related attenuation of vascular uptake would be expected to affect both approaches within the same PET/CT examination and is unlikely to explain the observed difference between visual and semiquantitative assessment.

In subgroup analyses, visual diagnostic accuracy remained broadly comparable in patients with and without glucocorticoid treatment at the time of imaging, although these results must be interpreted cautiously because of the small subgroup sizes. Among the 10 GCA patients receiving glucocorticoids at the time of [¹⁸F]FDG PET/CT, 9 were identified as GCA by visual assessment, suggesting that treatment did not fully abolish visually detectable vascular uptake in this cohort.

### Limitations

This study has several limitations. First, as a single-center study, its generalizability is inherently limited and the retrospective design introduces the potential for inclusion bias. However, all patients at our institution who underwent [¹⁸F]FDG PET/CT for suspected GCA and had available consent and sufficient follow-up were included, which likely mitigates the impact of such bias on the validity of the results. It should be considered that the non-GCA group was not a healthy or predefined disease-control group, but consisted of patients with suspected GCA and alternative final diagnoses after expert chart review, reflecting the clinical population in which [^18^F]FDG PET/CT is used diagnostically.

Second, images were acquired using 3 different scanner models, and semiquantitative parameters derived from acquisitions on different scanners can be different despite identical FDG doses and patient characteristics [10]. However, because diagnostic accuracy was comparable across scanner models, this has unlikely relevantly affected the results in our cohort and may also partially simulate a multicenter setting.

Third, 46/163 patients, including 10/39 patients with GCA, were receiving glucocorticoids at the time of [¹⁸F]FDG PET/CT. This may have reduced vascular tracer uptake and could have led to underestimation of active inflammation. Importantly, all patients were referred for suspected new-onset GCA in the setting of clinically active disease rather than remission. Therefore, persistent vascular uptake in treated patients was interpreted in the context of ongoing clinical suspicion of active vasculitis. Nevertheless, glucocorticoid exposure may have attenuated vascular tracer uptake and could therefore have led to underestimation of inflammatory activity. Although subgroup analyses stratified by uptake time did not indicate a consistent impact on the comparative performance of visual and semiquantitative analysis, these results should be interpreted cautiously because of limited subgroup sizes. Nevertheless, the study aimed to compare visual and semiquantitative assessment within the same real-world cohort, and both methods were affected by this potential confounder to the same extent.

Fourth, uptake time after FDG injection varied (60, 90, or 120 min). Longer uptake times were used because they have been shown to increase sensitivity for suspected LVV, particularly after initiation of glucocorticoid therapy [20, 21] However, this variability complicates the use of fixed SUV cut-off values. As longer uptake times are associated with higher vascular wall SUVs, lower blood-pool activity, and largely stable liver uptake, SUV ratios would be expected to perform better rather than worse in this setting [21–23].

Fifth, the diagnostic reference standard was derived from chart review, precluding complete blinding to the original [^18^F]FDG PET/CT results for the clinical investigators establishing the final diagnosis. This may have introduced incorporation bias, as PET/CT findings were part of routine clinical care and may have contributed to the clinical diagnosis. However, all PET/CT scans were re-read for the present analysis by readers blinded to clinical information and to the original PET/CT reports, the imaging findings documented in the original clinical reports were not used in any analysis, and visual and semiquantitative methods were compared within the same patients. In addition, the final diagnosis was based on at least 6 months of follow-up and on additional diagnostic data, including results from temporal artery biopsy and other imaging studies. These measures likely limited, although they cannot fully eliminate, potential incorporation bias.

Finally, the proportion of patients with GCA was relatively low (24%) compared with other of our institutional cohorts (> 50%), reflecting our preferential use of [¹⁸F]FDG PET/CT only in diagnostically challenging cases where other imaging modalities (ultrasound or MR) were inconclusive, not available or not possible to perform due to contraindication [24].

## Conclusion

In conclusion, our study supports the use of visual analysis of [¹⁸F]FDG PET/CT in patients with suspected GCA or LVV, due to its generalizability, advantages in diagnostic accuracy and ease of application in clinical practice. Based on our data, the use of semiquantitative parameters should not be recommended.

## Supplementary Information

Below is the link to the electronic supplementary material.

Supplementary Material 1 (DOCX 668 KB)

## Data Availability

The datasets generated during the current study are available from the corresponding author on reasonable request.

## Data availability

The data underlying this article will be shared on reasonable request to the corresponding author.
