# Supplementary material for: Visual analysis of [18F]FDG PET/CT imaging yields superior diagnostic accuracy compared to semiquantitative analysis in the assessment of giant cell arteritis
Source: Eur J Nucl Med Mol Imaging. 2026 Jun 6;53(10):6063–74. doi: 10.1007/s00259-026-07981-5 (PMC13652860; doi:10.1007/s00259-026-07981-5)
Supplement: Supplementary file 1 [file 259_2026_7981_MOESM1_ESM.docx]

**Supplementary material**

**Manuscript:**

Luca Seitz^1^, Rahel Friedli^1^, Nasir Gözlügöl^2^, Susana Bucher^1^, Pascal Seitz^1^, Fabian Lötscher^1^, Marc Gilgen^1^, Jennifer Amsler^1,3^, Patrick Stähli^4^, Fabrice Helfenstein^4,5^, Yvonne Döring^4,6,7,8^, Györgyi Hamvas^4^, Marc Schindewolf^4^, Axel Rominger^2^, Britta Maurer^1^, Lisa Christ^1^, Federico Caobelli^2^

1 Department of Rheumatology and Immunology, Inselspital, University Hospital Bern, University of Bern, Bern, Switzerland

2 Department of Nuclear Medicine, Inselspital, Bern University Hospital, University of Bern, Bern, Switzerland

3 Graduate School for Health Sciences, University of Bern, Switzerland

4 Department of Angiology, Inselspital, University Hospital Bern, University of Bern, Bern, Switzerland

5 CTU Bern, Department of Clinical Research, University of Bern, Bern, Switzerland

6 Institute for Cardiovascular Prevention (IPEK), Ludwig-Maximilians-University Munich, Germany

7 DZHK (German Center for Cardiovascular Research), Partner Site Munich Heart Alliance, Germany

8 Department for BioMedical Research (DBMR), University of Bern, Bern, Switzerland

| **Artery subgroup** | **Arteries** |
| --- | --- |
| Head | temporal artery, occipital artery, facial artery |
| Neck | vertebral artery, common carotid artery, internal carotid artery, external carotid artery |
| Arm | subclavian artery, axillary artery, brachial artery |
| Thorax | innominate artery, ascending aorta, aortic arch, descending thoracic aorta |
| Neck-Arm | vertebral artery, common carotid artery, internal carotid artery, external carotid artery; subclavian artery, axillary artery, brachial artery |
| Neck-Arm-Thorax | vertebral artery, common carotid artery, internal carotid artery, external carotid artery; subclavian artery, axillary artery, brachial artery; innominate artery, ascending aorta, aortic arch, descending thoracic aorta |
| Abdomen-Pelvis | abdominal aorta, common iliac artery, internal iliac artery, external iliac artery, femoral artery |

**Supplementary Table 1. Subgroup of arteries used for cut-off derivations on subgroup levels.**

| **Characteristic** | **Total Population**  **(N = 163) ^a^** | **GCA**  **(N=39) ^a^** | **Non-GCA**  **(N=124) ^a^** | **p-value** |
| --- | --- | --- | --- | --- |
| **Liver** |  |  |  |  |
| **SUVmax** | 3.071 (0.689) \| 2.930  [1.800; 2.570;3.540; 5.450] | 2.926 (0.593) \| 2.850 [2.100; 2.470;3.250; 4.860] | 3.117 (0.712) \| 3.040 [1.800; 2.630;3.645; 5.450] | 0.10 |
| **SUVmean** | 2.190 (0.470) \| 2.150  [1.060; 1.880;2.470; 4.250] | 2.036 (0.315) \| 2.080 [1.390; 1.850;2.230; 2.720] | 2.238 (0.500) \| 2.210 [1.060; 1.890;2.540; 4.250] | 0.004 |
| **SUVpeak** | 2.533 (0.564) \| 2.480  [1.420; 2.160;2.800; 4.900] | 2.346 (0.387) \| 2.320 [1.720; 2.080;2.560; 3.650] | 2.592 (0.598) \| 2.575 [1.420; 2.240;2.915; 4.900] | 0.003 |
| **Blood pool** |  |  |  |  |
| **SUVmax** | 2.123 (0.518) \| 2.070  [1.020; 1.770;2.490; 3.900] | 1.904 (0.394) \| 1.900 [1.080; 1.690;2.090; 3.220] | 2.192 (0.535) \| 2.205 [1.020; 1.795;2.555; 3.900] | <0.001 |
| **SUVmean** | 1.568 (0.389) \| 1.550  [0.580; 1.290;1.790; 3.130] | 1.427 (0.304) \| 1.360 [0.910; 1.190;1.630; 2.130] | 1.612 (0.403) \| 1.575 [0.580; 1.340;1.850; 3.130] | 0.003 |
| **SUVpeak** | 1.855 (0.436) \| 1.800  [0.720; 1.550;2.140; 3.800] | 1.677 (0.339) \| 1.690 [1.000; 1.410;1.860; 2.640] | 1.911 (0.448) \| 1.890 [0.720; 1.615;2.225; 3.800] | <0.001 |

**Supplementary Table 2. Standardized uptake values (SUVs) from the liver and blood pool.** GCA, giant cell arteritis; Q, quartile; SUV, standardized uptake value. a, Mean (SD) | Median [minimum; 25^th^ percentile; 75^th^ percentile; maximum].

| **Parameter** | **Youden`s threshold** | **Sensitivity** | **Specificity** | **Accuracy** | **PPV** | **NPV** | **Positive LR** | **Negative LR** |
| --- | --- | --- | --- | --- | --- | --- | --- | --- |
| **SUVmax Head** | 1.625 | 0.359 (0.212-0.528) | 0.831 (0.753-0.892) | 0.718 (0.642-0.785) | 0.400 (0.239-0.579) | 0.805 (0.725-0.869) | 2.120 (1.196-3.758) | 0.772 (0.602-0.989) |
| SUVpeak Head | 0.695 | 0.744 (0.579-0.87) | 0.218 (0.149-0.301) | 0.344 (0.271-0.422) | 0.230 (0.160-0.314) | 0.730 (0.559-0.862) | 0.951 (0.773-1.168) | 1.178 (0.627-2.211) |
| SUVmean Head | 1.145 | 0.308 (0.17-0.476) | 0.823 (0.744-0.885) | 0.699 (0.623-0.769) | 0.353 (0.197-0.535) | 0.791 (0.710-0.857) | 1.734 (0.948-3.174) | 0.842 (0.672-1.054) |
| Head: Artery SUVmax / Liver SUVmax | 0.631 | 0.333 (0.191-0.502) | 0.903 (0.837-0.949) | 0.767 (0.694-0.829) | 0.520 (0.313-0.722) | 0.812 (0.736-0.873) | 3.444 (1.715-6.917) | 0.738 (0.587-0.928) |
| Head: Artery SUVmax / Liver SUVmean | 0.821 | 0.333 (0.191-0.502) | 0.871 (0.799-0.924) | 0.742 (0.668-0.808) | 0.448 (0.264-0.643) | 0.806 (0.729-0.869) | 2.583 (1.366-4.886) | 0.765 (0.607-0.965) |
| Head: Artery SUVmean / Liver SUVmean | 0.399 | 0.59 (0.421-0.744) | 0.605 (0.513-0.691) | 0.601 (0.522-0.677) | 0.319 (0.214-0.440) | 0.824 (0.730-0.896) | 1.492 (1.062-2.098) | 0.678 (0.454-1.014) |
| Head: Artery SUVpeak / Liver SUVpeak | 0.497 | 0.436 (0.278-0.604) | 0.742 (0.656-0.816) | 0.669 (0.591-0.740) | 0.347 (0.217-0.496) | 0.807 (0.723-0.875) | 1.689 (1.061-2.690) | 0.760 (0.566-1.021) |
| Head: Artery SUVmax / Blood SUVmax | 0.724 | 0.513 (0.348-0.676) | 0.774 (0.690-0.844) | 0.712 (0.636-0.780) | 0.417 (0.276-0.568) | 0.835 (0.754-0.897) | 2.271 (1.452-3.551) | 0.629 (0.450-0.880) |
| Head: Artery SUVmax / Blood SUVmean | 0.86 | 0.615 (0.446-0.766) | 0.621 (0.529-0.707) | 0.620 (0.540-0.694) | 0.338 (0.230-0.460) | 0.837 (0.745-0.906) | 1.624 (1.161-2.270) | 0.619 (0.407-0.943) |
| Head: Artery SUVmean / Blood SUVmean | 0.509 | 0.667 (0.498-0.809) | 0.508 (0.417-0.599) | 0.546 (0.466-0.624) | 0.299 (0.205-0.406) | 0.829 (0.725-0.906) | 1.355 (1.019-1.802) | 0.656 (0.407-1.057) |
| Head: Artery SUVpeak / Blood SUVpeak | 0.63 | 0.462 (0.301-0.628) | 0.694 (0.604-0.773) | 0.638 (0.559-0.712) | 0.321 (0.203-0.460) | 0.804 (0.716-0.874) | 1.506 (0.980-2.316) | 0.776 (0.568-1.062) |
| **SUVmax Abdominal-Pelvic** | 2.455 | 0.795 (0.635-0.907) | 0.524 (0.433-0.615) | 0.589 (0.509-0.665) | 0.344 (0.247-0.452) | 0.890 (0.795-0.951) | 1.671 (1.309-2.132) | 0.391 (0.206-0.742) |
| SUVpeak Abdominal-Pelvic | 2.305 | 0.513 (0.348-0.676) | 0.758 (0.673-0.830) | 0.699 (0.623-0.769) | 0.400 (0.264-0.548) | 0.832 (0.750-0.896) | 2.120 (1.370-3.280) | 0.643 (0.459-0.900) |
| SUVmean Abdominal-Pelvic | 2.205 | 0.513 (0.348-0.676) | 0.823 (0.744-0.885) | 0.748 (0.675-0.813) | 0.476 (0.320-0.636) | 0.843 (0.766-0.903) | 2.890 (1.776-4.704) | 0.592 (0.425-0.826) |
| Abdominal-Pelvic: Artery SUVmax / Liver SUVmax | 0.961 | 0.641 (0.472-0.788) | 0.823 (0.744-0.885) | 0.779 (0.708-0.840) | 0.532 (0.381-0.679) | 0.879 (0.806-0.932) | 3.613 (2.313-5.643) | 0.436 (0.285-0.669) |
| Abdominal-Pelvic: Artery SUVmax / Liver SUVmean | 1.066 | 0.923 (0.791-0.984) | 0.435 (0.347-0.527) | 0.552 (0.472-0.630) | 0.340 (0.250-0.438) | 0.947 (0.854-0.989) | 1.635 (1.367-1.956) | 0.177 (0.058-0.534) |
| Abdominal-Pelvic: Artery SUVmean / Liver SUVmean | 0.855 | 0.821 (0.665-0.925) | 0.524 (0.433-0.615) | 0.595 (0.516-0.671) | 0.352 (0.254-0.459) | 0.903 (0.810-0.960) | 1.724 (1.362-2.183) | 0.342 (0.171-0.684) |
| Abdominal-Pelvic: Artery SUVpeak / Liver SUVpeak | 0.801 | 0.821 (0.665-0.925) | 0.500 (0.409-0.591) | 0.577 (0.497-0.654) | 0.340 (0.246-0.445) | 0.899 (0.802-0.958) | 1.641 (1.305-2.064) | 0.359 (0.179-0.718) |
| Abdominal-Pelvic: Artery SUVmax / Blood SUVmax | 1.228 | 0.846 (0.695-0.941) | 0.637 (0.546-0.722) | 0.687 (0.610-0.757) | 0.423 (0.312-0.540) | 0.929 (0.853-0.974) | 2.332 (1.782-3.051) | 0.241 (0.114-0.510) |
| Abdominal-Pelvic: Artery SUVmax / Blood SUVmean | 1.541 | 0.872 (0.726-0.957) | 0.500 (0.409-0.591) | 0.589 (0.509-0.665) | 0.354 (0.259-0.458) | 0.925 (0.834-0.975) | 1.744 (1.409-2.158) | 0.256 (0.111-0.592) |
| Abdominal-Pelvic: Artery SUVmean / Blood SUVmean | 1.17 | 0.795 (0.635-0.907) | 0.500 (0.409-0.591) | 0.571 (0.491-0.648) | 0.333 (0.239-0.439) | 0.886 (0.787-0.949) | 1.590 (1.254-2.016) | 0.410 (0.216-0.780) |
| Abdominal-Pelvic: Artery SUVpeak / Blood SUVpeak | 1.132 | 0.769 (0.607-0.889) | 0.597 (0.505-0.684) | 0.638 (0.559-0.712) | 0.375 (0.269-0.490) | 0.892 (0.804-0.949) | 1.908 (1.450-2.511) | 0.387 (0.214-0.698) |
| **SUVmax Neck-Arm-Thorax** | 2.525 | 0.821 (0.665-0.925) | 0.516 (0.425-0.607) | 0.589 (0.509-0.665) | 0.348 (0.251-0.454) | 0.901 (0.807-0.959) | 1.696 (1.342-2.142) | 0.348 (0.174-0.695) |
| SUVpeak Neck-Arm-Thorax | 2.225 | 0.769 (0.607-0.889) | 0.661 (0.571-0.744) | 0.687 (0.610-0.757) | 0.417 (0.302-0.539) | 0.901 (0.821-0.954) | 2.271 (1.682-3.066) | 0.349 (0.194-0.627) |
| SUVmean Neck-Arm-Thorax | 1.975 | 0.769 (0.607-0.889) | 0.540 (0.449-0.630) | 0.595 (0.516-0.671) | 0.345 (0.246-0.454) | 0.882 (0.787-0.944) | 1.673 (1.294-2.163) | 0.427 (0.235-0.775) |
| Neck-Arm-Thorax: Artery SUVmax / Liver SUVmax | 0.808 | 0.897 (0.758-0.971) | 0.411 (0.324-0.503) | 0.528 (0.448-0.606) | 0.324 (0.237-0.421) | 0.927 (0.824-0.980) | 1.524 (1.272-1.828) | 0.249 (0.096-0.646) |
| Neck-Arm-Thorax: Artery SUVmax / Liver SUVmean | 1.085 | 0.949 (0.827-0.994) | 0.363 (0.278-0.454) | 0.503 (0.424-0.582) | 0.319 (0.236-0.412) | 0.957 (0.855-0.995) | 1.489 (1.280-1.733) | 0.141 (0.036-0.556) |
| Neck-Arm-Thorax: Artery SUVmean / Liver SUVmean | 0.867 | 0.872 (0.726-0.957) | 0.476 (0.385-0.567) | 0.571 (0.491-0.648) | 0.343 (0.251-0.446) | 0.922 (0.827-0.974) | 1.663 (1.353-2.044) | 0.269 (0.116-0.624) |
| Neck-Arm-Thorax: Artery SUVpeak / Liver SUVpeak | 0.823 | 0.821 (0.665-0.925) | 0.492 (0.401-0.583) | 0.571 (0.491-0.648) | 0.337 (0.243-0.441) | 0.897 (0.799-0.958) | 1.615 (1.287-2.027) | 0.365 (0.182-0.731) |
| Neck-Arm-Thorax: Artery SUVmax / Blood SUVmax | 1.173 | 0.897 (0.758-0.971) | 0.406 (0.370-0.551) | 0.564 (0.485-0.642) | 0.343 (0.252-0.444) | 0.934 (0.841-0.982) | 1.661 (1.368-2.016) | 0.223 (0.086-0.576) |
| Neck-Arm-Thorax: Artery SUVmax / Blood SUVmean | 1.456 | 0.949 (0.827-0.994) | 0.355 (0.271-0.446) | 0.497 (0.418-0.576) | 0.316 (0.233-0.409) | 0.957 (0.852-0.995) | 1.471 (1.266-1.708) | 0.145 (0.037-0.569) |
| Neck-Arm-Thorax: Artery SUVmean / Blood SUVmean | 1.176 | 0.923 (0.791-0.984) | 0.476 (0.385-0.567) | 0.583 (0.503-0.659) | 0.356 (0.264-0.458) | 0.952 (0.865-0.990) | 1.761 (1.455-2.131) | 0.162 (0.054-0.487) |
| Neck-Arm-Thorax: Artery SUVpeak / Blood SUVpeak | 1.07 | 0.872 (0.726-0.957) | 0.435 (0.347-0.527) | 0.540 (0.460-0.618) | 0.327 (0.238-0.426) | 0.915 (0.813-0.972) | 1.544 (1.270-1.879) | 0.294 (0.127-0.684) |

**Supplementary Table 3. Measures of diagnostic accuracy using cut-offs derived from ROC at subgroup levels (all 163 patients):** Values in parentheses are 95%-confidence intervals. LR, likelihood ratio; NPV, negative predictive value; PPV, positive predictive value; SUV, standardized uptake value.

| **Parameter** | **Threshold**  **(Youden)** | **AUC** | **Sensitivity** | **Specificity** | **Accuracy** | **PPV** | **NPV** | **Positive LR** | **Negative LR** |
| --- | --- | --- | --- | --- | --- | --- | --- | --- | --- |
| Artery SUVmax | 2.525 | 0.625 (0.602-0.647) | 0.897 (0.758-0.971) | 0.403 (0.316-0.495) | 0.521 (0.442-0.600) | 0.321 (0.235-0.417) | 0.926 (0.821-0.979) | 1.504 (1.257-1.799) | 0.254 (0.098-0.660) |
| Artery SUVmean | 1.975 | 0.601 (0.579-0.623) | 0.821 (0.665-0.925) | 0.476 (0.385-0.567) | 0.558 (0.479-0.636) | 0.330 (0.238-0.433) | 0.894 (0.794-0.956) | 1.565 (1.253-1.956) | 0.377 (0.188-0.757) |
| Artery SUVpeak | 2.225 | 0.599 (0.577-0.622) | 0.821 (0.665-0.925) | 0.573 (0.481-0.661) | 0.632 (0.553-0.706) | 0.376 (0.274-0.488) | 0.910 (0.824-0.963) | 1.920 (1.493-2.468) | 0.313 (0.158-0.624) |
| artery SUVmax/liver SUVmax | 0.917 | 0.652 (0.630-0.674) | 0.872 (0.726-0.957) | 0.589 (0.497-0.676) | 0.656 (0.578-0.729) | 0.400 (0.295-0.512) | 0.936 (0.857-0.979) | 2.120 (1.663-2.701) | 0.218 (0.095-0.500) |
| artery SUVmax/liver SUVmean | 1.081 | 0.682 (0.661-0.704) | 0.974 (0.865-0.999) | 0.290 (0.212-0.379) | 0.454 (0.376-0.534) | 0.302 (0.223-0.390) | 0.973 (0.858-0.999) | 1.373 (1.213-1.554) | 0.088 (0.013-0.623) |
| artery SUVmean/liver SUVmean | 0.862 | 0.655 (0.633-0.676) | 0.923 (0.791-0.984) | 0.371 (0.286-0.462) | 0.503 (0.424-0.582) | 0.316 (0.232-0.409) | 0.939 (0.831-0.987) | 1.467 (1.247-1.727) | 0.207 (0.068-0.630) |
| artery SUVpeak/liver SUVpeak | 0.801 | 0.660 (0.638-0.682) | 0.897 (0.758-0.971) | 0.371 (0.286-0.462) | 0.497 (0.418-0.576) | 0.310 (0.226-0.404) | 0.920 (0.808-0.978) | 1.427 (1.201-1.694) | 0.276 (0.106-0.719) |
| artery SUVmax/blood SUVmax | 1.229 | 0.704 (0.683-0.725) | 0.949 (0.827-0.994) | 0.484 (0.393-0.575) | 0.595 (0.516-0.671) | 0.366 (0.273-0.468) | 0.968 (0.888-0.996) | 1.838 (1.527-2.213) | 0.106 (0.027-0.414) |
| artery SUVmax/blood SUVmean | 1.541 | 0.697 (0.676-0.718) | 0.949 (0.827-0.994) | 0.387 (0.301-0.479) | 0.521 (0.442-0.600) | 0.327 (0.242-0.422) | 0.960 (0.863-0.995) | 1.548 (1.322-1.812) | 0.132 (0.034-0.520) |
| artery SUVmean/blood SUVmean | 1.175 | 0.671 (0.649-0.692) | 0.974 (0.865-0.999) | 0.403 (0.316-0.495) | 0.540 (0.460-0.618) | 0.339 (0.253-0.435) | 0.980 (0.896-1.000) | 1.633 (1.401-1.903) | 0.064 (0.009-0.445) |
| artery SUVpeak/blood SUVpeak | 1.07 | 0.690 (0.669-0.711) | 0.923 (0.791-0.984) | 0.355 (0.271-0.446) | 0.491 (0.412-0.570) | 0.310 (0.228-0.403) | 0.936 (0.825-0.987) | 1.431 (1.221-1.677) | 0.217 (0.071-0.660) |

**Supplementary Table 4. Cut-off determination derived from ROC from all arteries combined (3922 segments in all 163 patients) with the clinical diagnosis as reference:** Values in parentheses are 95%-confidence intervals. LR, likelihood ratio; NPV, negative predictive value; n.a., not applicable; PPV, positive predictive value; SUV, standardized uptake value.

| **Parameter** | **Threshold (Youden)** | **AUC** | **Sensitivity** | **Specificity** | **Accuracy** | **PPV** | **NPV** | **Positive LR** | **Negative LR** |
| --- | --- | --- | --- | --- | --- | --- | --- | --- | --- |
| Artery SUVmax | 2.525 | 0.632 (0.609-0.656) | 0.406 (0.373-0.439) | 0.869 (0.855-0.881) | 0.758 (0.743-0.771) | 0.494 (0.457-0.531) | 0.822 (0.808-0.836) | 3.088 (2.725-3.499) | 0.684 (0.646-0.724) |
| Artery SUVmean | 1.975 | 0.608 (0.585-0.631) | 0.302 (0.271-0.333) | 0.896 (0.884-0.907) | 0.753 (0.739-0.767) | 0.477 (0.435-0.520) | 0.802 (0.788-0.816) | 2.891 (2.492-3.355) | 0.780 (0.745-0.816) |
| Artery SUVpeak | 2.225 | 0.607 (0.584-0.631) | 0.288 (0.258-0.319) | 0.925 (0.915-0.935) | 0.772 (0.758-0.786) | 0.548 (0.501-0.594) | 0.804 (0.790-0.818) | 3.839 (3.247-4.540) | 0.770 (0.737-0.804) |
| artery SUVmax/liver SUVmax | 0.808 | 0.659 (0.636-0.683) | 0.464 (0.431-0.498) | 0.831 (0.816-0.845) | 0.743 (0.728-0.757) | 0.464 (0.431-0.498) | 0.831 (0.816-0.845) | 2.746 (2.462-3.063) | 0.645 (0.605-0.687) |
| artery SUVmax/liver SUVmean | 1.113 | 0.693 (0.671-0.715) | 0.476 (0.442-0.510) | 0.843 (0.829-0.857) | 0.755 (0.741-0.769) | 0.489 (0.455-0.524) | 0.836 (0.822-0.849) | 3.035 (2.716-3.391) | 0.622 (0.582-0.663) |
| artery SUVmean/liver SUVmean | 0.862 | 0.665 (0.642-0.687) | 0.396 (0.363-0.429) | 0.877 (0.864-0.889) | 0.762 (0.747-0.775) | 0.504 (0.466-0.542) | 0.821 (0.807-0.835) | 3.222 (2.832-3.666) | 0.689 (0.652-0.728) |
| artery SUVpeak/liver SUVpeak | 0.801 | 0.67 (0.648-0.693) | 0.450 (0.416-0.483) | 0.842 (0.828-0.855) | 0.748 (0.733-0.762) | 0.473 (0.438-0.507) | 0.829 (0.814-0.843) | 2.840 (2.536-3.180) | 0.654 (0.615-0.696) |
| artery SUVmax/blood SUVmax | 1.229 | 0.713 (0.692-0.735) | 0.478 (0.445-0.512) | 0.870 (0.856-0.882) | 0.776 (0.762-0.789) | 0.537 (0.501-0.572) | 0.841 (0.827-0.854) | 3.668 (3.257-4.130) | 0.600 (0.562-0.640) |
| artery SUVmax/blood SUVmean | 1.441 | 0.708 (0.687-0.73) | 0.592 (0.558-0.625) | 0.755 (0.739-0.771) | 0.716 (0.701-0.731) | 0.433 (0.405-0.462) | 0.854 (0.840-0.868) | 2.417 (2.219-2.633) | 0.541 (0.498-0.587) |
| arterySUVmean/blood SUVmean | 1.175 | 0.682 (0.66-0.703) | 0.447 (0.414-0.481) | 0.846 (0.832-0.859) | 0.750 (0.736-0.764) | 0.479 (0.444-0.513) | 0.829 (0.815-0.843) | 2.906 (2.591-3.258) | 0.653 (0.614-0.695) |
| artery SUVpeak/blood SUVpeak | 1.07 | 0.702 (0.681-0.724) | 0.506 (0.472-0.539) | 0.827 (0.812-0.840) | 0.750 (0.735-0.764) | 0.479 (0.447-0.512) | 0.841 (0.827-0.855) | 2.915 (2.626-3.236) | 0.598 (0.558-0.641) |

**Supplementary Table 5. Cut-off determination derived from ROC from arteries of neck/thorax/pelvis/thigh combined (3633 segments in all 163 patients) with the clinical diagnosis as reference:** Values in parentheses are 95%-confidence intervals. LR, likelihood ratio; NPV, negative predictive value; n.a., not applicable; PPV, positive predictive value; SUV, standardized uptake value.

| **Artery subgroup SUV/Ratio** | **N (patients, segments)** | **AUC** | **Prevalence** | **Threshold (Youden)** | **Sensitivity** | **Specificity** | **Accuracy** | **PPV** | **NPV** | **Positive LR** | **Negative LR** |
| --- | --- | --- | --- | --- | --- | --- | --- | --- | --- | --- | --- |
| SUVmax  Head | 138 patients/289 segments | 0.580 (0.494-0.667) | 0.246 (0.197-0.299) | 1.625 | 0.366 (0.255-0.489) | 0.862 (0.809-0.905) | 0.740 (0.686-0.790) | 0.464 (0.330-0.603) | 0.807 (0.750-0.856) | 2.661 (1.694-4.181) | 0.735 (0.611-0.884) |
| SUVpeak  Head | 138 patients/289 segments | 0.482 (0.397-0.567) | 0.246 (0.197-0.299) | 0.695 | 0.197 (0.112-0.309) | 0.881 (0.830-0.921) | 0.713 (0.657-0.764) | 0.350 (0.206-0.517) | 0.771 (0.714-0.822) | 1.653 (0.915-2.988) | 0.912 (0.804-1.033) |
| SUVmean  Head | 138 patients/289 segments | 0.555 (0.471-0.638) | 0.246 (0.197-0.299) | 1.145 | 0.282 (0.181-0.401) | 0.872 (0.820-0.913) | 0.727 (0.671-0.777) | 0.417 (0.276-0.568) | 0.788 (0.731-0.838) | 2.193 (1.320-3.643) | 0.824 (0.706-0.962) |
| SUVmax  Neck-Arm | 163 patients/1545 segments | 0.641 (0.605-0.677) | 0.244 (0.223-0.266) | 2.66 | 0.342 (0.294-0.392) | 0.961 (0.949-0.972) | 0.810 (0.790-0.830) | 0.741 (0.670-0.805) | 0.819 (0.798-0.839) | 8.881 (6.457-12.217) | 0.684 (0.636-0.737) |
| SUVpeak  Neck-Arm | 163 patients/1545 segments | 0.634 (0.598-0.669) | 0.244 (0.223-0.266) | 2.045 | 0.334 (0.287-0.384) | 0.938 (0.922-0.951) | 0.790 (0.769-0.810) | 0.633 (0.562-0.700) | 0.814 (0.792-0.834) | 5.347 (4.107-6.962) | 0.710 (0.660-0.764) |
| SUVmean  Neck-Arm | 163 patients/1545 segments | 0.630 (0.594-0.665) | 0.244 (0.223-0.266) | 1.975 | 0.334 (0.287-0.384) | 0.934 (0.918-0.948) | 0.788 (0.766-0.808) | 0.621 (0.550-0.688) | 0.813 (0.791-0.833) | 5.070 (3.914-6.566) | 0.713 (0.663-0.767) |
| SUVmax  Thorax | 163 patients/640 segments | 0.666 (0.612-0.720) | 0.234 (0.202-0.269) | 2.525 | 0.567 (0.483-0.647) | 0.743 (0.702-0.781) | 0.702 (0.664-0.737) | 0.403 (0.336-0.472) | 0.848 (0.811-0.881) | 2.204 (1.794-2.706) | 0.583 (0.482-0.706) |
| SUVpeak  Thorax | 163 patients/640 segments | 0.623 (0.567-0.679) | 0.234 (0.202-0.269) | 2.225 | 0.407 (0.327-0.490) | 0.847 (0.812-0.878) | 0.744 (0.708-0.777) | 0.449 (0.363-0.536) | 0.823 (0.787-0.856) | 2.657 (2.000-3.530) | 0.701 (0.610-0.804) |
| SUVmean  Thorax | 163 patients/640 segments | 0.614 (0.560-0.668) | 0.234 (0.202-0.269) | 1.555 | 0.660 (0.578-0.735) | 0.535 (0.489-0.580) | 0.564 (0.525-0.603) | 0.303 (0.253-0.356) | 0.837 (0.791-0.876) | 1.418 (1.222-1.646) | 0.636 (0.501-0.807) |
| SUVmax  Abdominal-Pelvic | 163 patients/1448 segments | 0.610 (0.573-0.647) | 0.238 (0.217-0.261) | 2.455 | 0.409 (0.356-0.463) | 0.832 (0.809-0.854) | 0.731 (0.708-0.754) | 0.433 (0.378-0.488) | 0.818 (0.794-0.840) | 2.437 (2.030-2.925) | 0.710 (0.648-0.779) |
| SUVpeak  Abdominal-Pelvic | 163 patients/1448 segments | 0.572 (0.536-0.608) | 0.238 (0.217-0.261) | 2.305 | 0.209 (0.167-0.255) | 0.937 (0.921-0.951) | 0.764 (0.741-0.785) | 0.511 (0.425-0.596) | 0.791 (0.768-0.813) | 3.336 (2.454-4.536) | 0.844 (0.798-0.893) |
| SUVmean  Abdominal-Pelvic | 163 patients/1448 segments | 0.583 (0.548-0.619) | 0.238 (0.217-0.261) | 2.205 | 0.209 (0.167-0.255) | 0.949 (0.935-0.961) | 0.773 (0.750-0.794) | 0.562 (0.472-0.650) | 0.793 (0.770-0.815) | 4.111 (2.962-5.704) | 0.834 (0.788-0.882) |
| SUVmax  Neck | 163 patients/699 segments | 0.628 (0.575-0.682) | 0.253 (0.221-0.287) | 2.66 | 0.350 (0.280-0.425) | 0.950 (0.928-0.967) | 0.798 (0.767-0.827) | 0.705 (0.598-0.797) | 0.812 (0.778-0.842) | 7.033 (4.598-10.757) | 0.684 (0.613-0.763) |
| SUVpeak  Neck | 163 patients/699 segments | 0.611 (0.558-0.664) | 0.253 (0.221-0.287) | 2.225 | 0.294 (0.228-0.367) | 0.954 (0.932-0.970) | 0.787 (0.755-0.817) | 0.684 (0.567-0.786) | 0.799 (0.766-0.830) | 6.390 (4.064-10.048) | 0.740 (0.672-0.816) |
| SUVmean  Neck | 163 patients/699 segments | 0.614 (0.561-0.666) | 0.253 (0.221-0.287) | 1.975 | 0.362 (0.291-0.437) | 0.891 (0.861-0.916) | 0.757 (0.723-0.788) | 0.529 (0.436-0.620) | 0.804 (0.770-0.836) | 3.311 (2.420-4.531) | 0.717 (0.639-0.804) |
| SUVmax  Arm | 163 patients/846 segments | 0.649 (0.600-0.698) | 0.236 (0.208-0.267) | 2.835 | 0.330 (0.265-0.400) | 0.981 (0.968-0.990) | 0.827 (0.800-0.852) | 0.846 (0.747-0.918) | 0.826 (0.797-0.852) | 17.765 (9.806-32.185) | 0.683 (0.619-0.753) |
| SUVpeak  Arm | 163 patients/846 segments | 0.641 (0.592-0.689) | 0.236 (0.208-0.267) | 2.065 | 0.340 (0.275-0.410) | 0.971 (0.954-0.982) | 0.822 (0.794-0.847) | 0.782 (0.680-0.863) | 0.826 (0.797-0.852) | 11.56 (7.130-18.742) | 0.680 (0.615-0.752) |
| SUVmean  Arm | 163 patients/846 segments | 0.637 (0.589-0.686) | 0.236 (0.208-0.267) | 1.96 | 0.315 (0.251-0.384) | 0.967 (0.951-0.980) | 0.813 (0.785-0.839) | 0.750 (0.644-0.838) | 0.820 (0.791-0.847) | 9.690 (6.070-15.468) | 0.708 (0.644-0.779) |
| SUVmax  Neck-Arm-Thorax | 163 patients/2185 segments | 0.646 (0.615-0.676) | 0.241 (0.223-0.260) | 2.525 | 0.419 (0.377-0.463) | 0.881 (0.864-0.896) | 0.769 (0.751-0.787) | 0.527 (0.478-0.576) | 0.827 (0.808-0.844) | 3.512 (2.978-4.141) | 0.659 (0.612-0.711) |
| SUVpeak  Neck-Arm-Thorax | 163 patients/2185 segments | 0.629 (0.599-0.659) | 0.241 (0.223-0.260) | 2.225 | 0.328 (0.288-0.370) | 0.934 (0.921-0.945) | 0.788 (0.770-0.805) | 0.611 (0.552-0.668) | 0.814 (0.796-0.831) | 4.948 (3.979-6.153) | 0.719 (0.677-0.765) |
| SUVmean  Neck-Arm-Thorax | 163 patients/2185 segments | 0.624 (0.595-0.654) | 0.241 (0.223-0.260) | 1.975 | 0.338 (0.297-0.380) | 0.899 (0.883-0.913) | 0.763 (0.745-0.781) | 0.514 (0.460-0.568) | 0.810 (0.792-0.828) | 3.333 (2.766-4.017) | 0.737 (0.692-0.785) |

**Supplementary Table 6. Cut-off determination for SUVmax/SUVmean/SUVpeak derived from ROC from different artery subgroups in all 163 patients (head 138 patients) with the clinical diagnosis as reference:** Values in parentheses are 95%-confidence intervals. LR, likelihood ratio; NPV, negative predictive value; n.a., not applicable; PPV, positive predictive value; SUV, standardized uptake value.

| **Artery subgroup SUV/Ratio** | **N (patients, segments)** | **AUC** | **Prevalence** | **Threshold (Youden)** | **Sensitivity** | **Specificity** | **Accuracy** | **PPV** | **NPV** | **Positive LR** | **Negative LR** |
| --- | --- | --- | --- | --- | --- | --- | --- | --- | --- | --- | --- |
| **Head: Artery SUVmax / Liver SUVmax** | 138 patients/289 segments | 0.617 (0.531-0.702) | 0.246 (0.197-0.299) | 0.631 | 0.324 (0.218-0.445) | 0.931 (0.889-0.961) | 0.782 (0.73-0.828) | 0.605 (0.434-0.76) | 0.809 (0.755-0.856) | 4.708 (2.603-8.517) | 0.726 (0.616-0.856) |
| Head: Artery SUVmax / Liver SUVmean | 138 patients/289 segments | 0.63 (0.549-0.712) | 0.246 (0.197-0.299) | 0.821 | 0.352 (0.242-0.475) | 0.899 (0.851-0.936) | 0.765 (0.711-0.812) | 0.532 (0.381-0.679) | 0.81 (0.755-0.857) | 3.489 (2.103-5.790) | 0.721 (0.604-0.86) |
| Head: Artery SUVmean / Liver SUVmean | 138 patients/289 segments | 0.61 (0.528-0.691) | 0.246 (0.197-0.299) | 0.399 | 0.563 (0.44-0.681) | 0.651 (0.584-0.714) | 0.630 (0.571-0.686) | 0.345 (0.259-0.439) | 0.821 (0.755-0.875) | 1.616 (1.229-2.125) | 0.670 (0.506-0.888) |
| Head: Artery SUVpeak / Liver SUVpeak | 138 patients/289 segments | 0.597 (0.513-0.682) | 0.246 (0.197-0.299) | 0.497 | 0.437 (0.319-0.56) | 0.812 (0.754-0.862) | 0.720 (0.664-0.771) | 0.431 (0.314-0.553) | 0.816 (0.758-0.865) | 2.322 (1.585-3.401) | 0.694 (0.560-0.860) |
| Head: Artery SUVmax / Blood SUVmax | 138 patients/289 segments | 0.678 (0.595-0.762) | 0.246 (0.197-0.299) | 0.724 | 0.549 (0.427-0.668) | 0.812 (0.754-0.862) | 0.747 (0.693-0.796) | 0.488 (0.374-0.602) | 0.847 (0.791-0.893) | 2.921 (2.064-4.133) | 0.555 (0.426-0.723) |
| Head: Artery SUVmax / Blood SUVmean | 138 patients/289 segments | 0.649 (0.566-0.732) | 0.246 (0.197-0.299) | 0.860 | 0.648 (0.525-0.758) | 0.674 (0.608-0.736) | 0.668 (0.610-0.722) | 0.393 (0.304-0.488) | 0.855 (0.793-0.904) | 1.989 (1.539-2.571) | 0.522 (0.376-0.725) |
| Head: Artery SUVmean / Blood SUVmean | 138 patients/289 segments | 0.635 (0.555-0.716) | 0.246 (0.197-0.299) | 0.509 | 0.690 (0.569-0.795) | 0.560 (0.491-0.627) | 0.592 (0.533-0.649) | 0.338 (0.262-0.421) | 0.847 (0.778-0.902) | 1.567 (1.263-1.945) | 0.554 (0.384-0.799) |
| Head: Artery SUVpeak / Blood SUVpeak | 138 patients/289 segments | 0.629 (0.547-0.711) | 0.246 (0.197-0.299) | 0.630 | 0.493 (0.372-0.614) | 0.757 (0.694-0.812) | 0.692 (0.635-0.745) | 0.398 (0.295-0.508) | 0.821 (0.761-0.871) | 2.028 (1.454-2.827) | 0.670 (0.526-0.853) |
| **Neck-Arm: Artery SUVmax / Liver SUVmax** | 163 patients/1545 segments | 0.669 (0.634-0.704) | 0.244 (0.223-0.266) | 0.815 | 0.408 (0.358-0.46) | 0.911 (0.893-0.927) | 0.788 (0.767-0.808) | 0.597 (0.534-0.657) | 0.827 (0.805-0.847) | 4.588 (3.682-5.717) | 0.649 (0.596-0.707) |
| Neck-Arm: Artery SUVmax / Liver SUVmean | 163 patients/1545 segments | 0.698 (0.664-0.731) | 0.244 (0.223-0.266) | 1.085 | 0.432 (0.382-0.484) | 0.892 (0.873-0.909) | 0.780 (0.758-0.800) | 0.564 (0.505-0.622) | 0.830 (0.808-0.850) | 4.008 (3.277-4.902) | 0.636 (0.581-0.696) |
| Neck-Arm: Artery SUVmean / Liver SUVmean | 163 patients/1545 segments | 0.682 (0.648-0.716) | 0.244 (0.223-0.266) | 0.867 | 0.390 (0.340-0.441) | 0.914 (0.897-0.930) | 0.786 (0.765-0.807) | 0.595 (0.531-0.657) | 0.823 (0.801-0.843) | 4.554 (3.633-5.709) | 0.667 (0.614-0.725) |
| Neck-Arm: Artery SUVpeak / Liver SUVpeak | 163 patients/1545 segments | 0.691 (0.657-0.724) | 0.244 (0.223-0.266) | 0.823 | 0.395 (0.346-0.447) | 0.927 (0.911-0.941) | 0.797 (0.776-0.817) | 0.637 (0.572-0.698) | 0.826 (0.804-0.846) | 5.431 (4.273-6.902) | 0.652 (0.600-0.709) |
| Neck-Arm: Artery SUVmax / Blood SUVmax) | 163 patients/1545 segments | 0.715 (0.682-0.747) | 0.244 (0.223-0.266) | 1.130 | 0.499 (0.447-0.55) | 0.873 (0.853-0.892) | 0.782 (0.760-0.802) | 0.560 (0.505-0.613) | 0.844 (0.822-0.864) | 3.935 (3.283-4.718) | 0.574 (0.518-0.636) |
| Neck-Arm: Artery SUVmax / Blood SUVmean | 163 patients/1545 segments | 0.706 (0.673-0.74) | 0.244 (0.223-0.266) | 1.429 | 0.533 (0.481-0.584) | 0.833 (0.810-0.854) | 0.760 (0.738-0.781) | 0.508 (0.457-0.558) | 0.847 (0.825-0.867) | 3.193 (2.724-3.744) | 0.560 (0.502-0.626) |
| Neck-Arm: Artery SUVmean / Blood SUVmean | 163 patients/1545 segments | 0.695 (0.662-0.728) | 0.244 (0.223-0.266) | 1.018 | 0.562 (0.511-0.613) | 0.763 (0.737-0.787) | 0.714 (0.691-0.736) | 0.434 (0.389-0.479) | 0.844 (0.820-0.865) | 2.371 (2.070-2.717) | 0.574 (0.509-0.646) |
| Neck-Arm: Artery SUVpeak / Blood SUVpeak | 163 patients/1545 segments | 0.716 (0.684-0.748) | 0.244 (0.223-0.266) | 1.070 | 0.469 (0.418-0.521) | 0.894 (0.875-0.911) | 0.790 (0.769-0.810) | 0.588 (0.530-0.644) | 0.839 (0.818-0.859) | 4.422 (3.628-5.391) | 0.594 (0.539-0.654) |
| T**horax: Artery SUVmax / Liver SUVmax** | 163 patients/640 segments | 0.704 (0.651-0.757) | 0.234 (0.202-0.269) | 0.912 | 0.527 (0.444-0.609) | 0.820 (0.783-0.853) | 0.752 (0.716-0.785) | 0.473 (0.395-0.552) | 0.850 (0.814-0.881) | 2.933 (2.301-3.738) | 0.577 (0.485-0.686) |
| Thorax: Artery SUVmax / Liver SUVmean | 163 patients/640 segments | 0.74 (0.692-0.788) | 0.234 (0.202-0.269) | 1.113 | 0.700 (0.620-0.772) | 0.688 (0.645-0.729) | 0.691 (0.653-0.726) | 0.407 (0.346-0.47) | 0.882 (0.846-0.913) | 2.242 (1.895-2.652) | 0.436 (0.339-0.561) |
| Thorax: Artery SUVmean / Liver SUVmean | 163 patients/640 segments | 0.677 (0.622-0.732) | 0.234 (0.202-0.269) | 0.779 | 0.640 (0.558-0.717) | 0.663 (0.620-0.705) | 0.658 (0.620-0.695) | 0.368 (0.309-0.429) | 0.858 (0.818-0.891) | 1.901 (1.599-2.259) | 0.543 (0.434-0.678) |
| Thorax: Artery SUVpeak / Liver SUVpeak | 163 patients/640 segments | 0.711 (0.656-0.766) | 0.234 (0.202-0.269) | 0.838 | 0.620 (0.537-0.698) | 0.759 (0.719-0.796) | 0.727 (0.690-0.761) | 0.441 (0.373-0.511) | 0.867 (0.831-0.898) | 2.575 (2.106-3.148) | 0.501 (0.406-0.618) |
| Thorax: Artery SUVmax / Blood SUVmax | 163 patients/640 segments | 0.748 (0.701-0.794) | 0.234 (0.202-0.269) | 1.211 | 0.633 (0.551-0.71) | 0.731 (0.689-0.769) | 0.708 (0.671-0.743) | 0.419 (0.354-0.486) | 0.867 (0.830-0.898) | 2.351 (1.944-2.843) | 0.502 (0.404-0.624) |
| Thorax: Artery SUVmax / Blood SUVmean | 163 patients/640 segments | 0.737 (0.689-0.784) | 0.234 (0.202-0.269) | 1.881 | 0.527 (0.444-0.609) | 0.843 (0.808-0.874) | 0.769 (0.734-0.801) | 0.506 (0.425-0.587) | 0.853 (0.819-0.884) | 3.352 (2.597-4.325) | 0.562 (0.472-0.668) |
| Thorax: Ratio 7 (Artery SUVmean / Blood SUVmean | 163 patients/640 segments | 0.676 (0.622-0.73) | 0.234 (0.202-0.269) | 1.289 | 0.467 (0.385-0.55) | 0.841 (0.805-0.872) | 0.753 (0.718-0.786) | 0.473 (0.390-0.557) | 0.837 (0.802-0.869) | 2.932 (2.247-3.824) | 0.634 (0.543-0.74) |
| Thorax: Artery SUVpeak / Blood SUVpeak | 163 patients/640 segments | 0.728 (0.677-0.779) | 0.234 (0.202-0.269) | 1.304 | 0.507 (0.424-0.589) | 0.882 (0.850-0.909) | 0.794 (0.760-0.824) | 0.567 (0.479-0.652) | 0.854 (0.820-0.883) | 4.280 (3.207-5.713) | 0.560 (0.474-0.66) |
| **Abdominal-Pelvic: Artery SUVmax / Liver SUVmax** | 163 patients/1448 segments | 0.638 (0.601-0.675) | 0.238 (0.217-0.261) | 0.961 | 0.313 (0.264-0.365) | 0.958 (0.945-0.969) | 0.805 (0.783-0.825) | 0.701 (0.622-0.772) | 0.817 (0.795-0.838) | 7.506 (5.433-10.37) | 0.717 (0.667-0.771) |
| Abdominal-Pelvic: Artery SUVmax / Liver SUVmean | 163 patients/1448 segments | 0.682 (0.647-0.717) | 0.238 (0.217-0.261) | 1.066 | 0.504 (0.450-0.558) | 0.803 (0.779-0.826) | 0.732 (0.708-0.755) | 0.445 (0.395-0.496) | 0.838 (0.815-0.86) | 2.564 (2.188-3.004) | 0.617 (0.553-0.689) |
| Abdominal-Pelvic: Artery SUVmean / Liver SUVmean | 163 patients/1448 segments | 0.646 (0.611-0.682) | 0.238 (0.217-0.261) | 0.855 | 0.362 (0.312-0.416) | 0.875 (0.854-0.894) | 0.753 (0.730-0.775) | 0.475 (0.414-0.538) | 0.814 (0.791-0.836) | 2.896 (2.348-3.571) | 0.729 (0.671-0.792) |
| Abdominal-Pelvic: Artery SUVpeak / Liver SUVpeak | 163 patients/1448 segments | 0.646 (0.611-0.682) | 0.238 (0.217-0.261) | 0.801 | 0.403 (0.351-0.457) | 0.834 (0.811-0.856) | 0.731 (0.708-0.754) | 0.432 (0.377-0.488) | 0.817 (0.793-0.839) | 2.428 (2.019-2.920) | 0.716 (0.654-0.784) |
| Abdominal-Pelvic: Artery SUVmax / Blood SUVmax | 163 patients/1448 segments | 0.708 (0.674-0.742) | 0.238 (0.217-0.261) | 1.228 | 0.472 (0.419-0.527) | 0.861 (0.839-0.881) | 0.769 (0.746-0.79) | 0.516 (0.459-0.572) | 0.839 (0.817-0.86) | 3.406 (2.832-4.096) | 0.612 (0.553-0.679) |
| Abdominal-Pelvic: Artery SUVmax / Blood SUVmean | 163 patients/1448 segments | 0.71 (0.676-0.743) | 0.238 (0.217-0.261) | 1.541 | 0.533 (0.479-0.587) | 0.813 (0.789-0.836) | 0.747 (0.723-0.769) | 0.472 (0.421-0.523) | 0.848 (0.825-0.869) | 2.856 (2.439-3.344) | 0.574 (0.511-0.645) |
| Abdominal-Pelvic: Artery SUVmean / Blood SUVmean | 163 patients/1448 segments | 0.675 (0.641-0.709) | 0.238 (0.217-0.261) | 1.17 | 0.423 (0.37-0.477) | 0.850 (0.828-0.871) | 0.749 (0.725-0.771) | 0.469 (0.413-0.527) | 0.825 (0.802-0.847) | 2.829 (2.346-3.411) | 0.678 (0.618-0.745) |
| Abdominal-Pelvic: Artery SUVpeak / Blood SUVpeak | 163 patients/1448 segments | 0.69 (0.657-0.724) | 0.238 (0.217-0.261) | 1.132 | 0.417 (0.365-0.471) | 0.873 (0.852-0.892) | 0.765 (0.742-0.786) | 0.507 (0.447-0.567) | 0.827 (0.804-0.849) | 3.288 (2.696-4.011) | 0.667 (0.609-0.732) |
| **Neck: Artery SUVmax / Liver SUVmax** | 163 patients/699 segments | 0.664 (0.612-0.716) | 0.253 (0.221-0.287) | 0.815 | 0.435 (0.361-0.511) | 0.875 (0.844-0.903) | 0.764 (0.731-0.795) | 0.542 (0.457-0.626) | 0.820 (0.786-0.851) | 3.494 (2.633-4.635) | 0.645 (0.565-0.737) |
| Neck: Artery SUVmax / Liver SUVmean | 163 patients/699 segments | 0.701 (0.652-0.751) | 0.253 (0.221-0.287) | 1.085 | 0.475 (0.399-0.551) | 0.872 (0.840-0.899) | 0.771 (0.738-0.802) | 0.556 (0.473-0.637) | 0.830 (0.796-0.861) | 3.697 (2.817-4.853) | 0.603 (0.522-0.696) |
| Neck: Artery SUVmean / Liver SUVmean | 163 patients/699 segments | 0.682 (0.632-0.731) | 0.253 (0.221-0.287) | 0.887 | 0.424 (0.350-0.500) | 0.879 (0.848-0.906) | 0.764 (0.731-0.795) | 0.543 (0.457-0.628) | 0.818 (0.784-0.849) | 3.511 (2.631-4.684) | 0.655 (0.575-0.747) |
| Neck: Artery SUVpeak / Liver SUVpeak | 163 patients/699 segments | 0.692 (0.642-0.741) | 0.253 (0.221-0.287) | 0.818 | 0.446 (0.372-0.523) | 0.875 (0.844-0.903) | 0.767 (0.734-0.798) | 0.549 (0.464-0.632) | 0.823 (0.789-0.854) | 3.584 (2.708-4.745) | 0.632 (0.552-0.725) |
| Neck: Artery SUVmax / Blood SUVmax | 163 patients/699 segments | 0.72 (0.671-0.769) | 0.253 (0.221-0.287) | 1.106 | 0.565 (0.489-0.639) | 0.833 (0.799-0.864) | 0.765 (0.732-0.796) | 0.535 (0.461-0.608) | 0.850 (0.816-0.879) | 3.390 (2.690-4.272) | 0.522 (0.439-0.62) |
| Neck: Artery SUVmax / Blood SUVmean | 163 patients/699 segments | 0.713 (0.664-0.761) | 0.253 (0.221-0.287) | 1.456 | 0.548 (0.472-0.623) | 0.822 (0.786-0.854) | 0.753 (0.719-0.784) | 0.511 (0.437-0.584) | 0.843 (0.808-0.873) | 3.076 (2.450-3.863) | 0.550 (0.465-0.65) |
| Neck: Artery SUVmean / Blood SUVmean | 163 patients/699 segments | 0.695 (0.647-0.743) | 0.253 (0.221-0.287) | 1.175 | 0.514 (0.438-0.59) | 0.824 (0.788-0.855) | 0.745 (0.711-0.777) | 0.497 (0.423-0.572) | 0.833 (0.798-0.864) | 2.917 (2.308-3.687) | 0.590 (0.504-0.69) |
| Neck: Artery SUVpeak / Blood SUVpeak | 163 patients/699 segments | 0.721 (0.674-0.768) | 0.253 (0.221-0.287) | 1.055 | 0.548 (0.472-0.623) | 0.810 (0.774-0.843) | 0.744 (0.710-0.776) | 0.495 (0.423-0.567) | 0.841 (0.806-0.872) | 2.890 (2.314-3.608) | 0.558 (0.472-0.659) |
| **Arm: Artery SUVmax / Liver SUVmax** | 163 patients/846 segments | 0.671 (0.623-0.719) | 0.236 (0.208-0.267) | 0.981 | 0.345 (0.279-0.415) | 0.986 (0.974-0.994) | 0.835 (0.808-0.859) | 0.885 (0.792-0.946) | 0.829 (0.801-0.855) | 24.763 (12.592-48.698) | 0.664 (0.600-0.735) |
| Arm: Artery SUVmax / Liver SUVmean | 163 patients/846 segments | 0.698 (0.653-0.743) | 0.236 (0.208-0.267) | 1.212 | 0.370 (0.303-0.441) | 0.966 (0.949-0.979) | 0.825 (0.798-0.85) | 0.771 (0.674-0.85) | 0.832 (0.803-0.858) | 10.865 (6.936-17.018) | 0.652 (0.586-0.726) |
| Arm: Artery SUVmean / Liver SUVmean | 163 patients/846 segments | 0.685 (0.639-0.73) | 0.236 (0.208-0.267) | 0.762 | 0.39 (0.322-0.461) | 0.918 (0.894-0.938) | 0.793 (0.764-0.82) | 0.595 (0.506-0.68) | 0.829 (0.800-0.856) | 4.754 (3.484-6.486) | 0.665 (0.593-0.744) |
| Arm: Artery SUVpeak / Liver SUVpeak | 163 patients/846 segments | 0.694 (0.649-0.739) | 0.236 (0.208-0.267) | 0.803 | 0.365 (0.298-0.436) | 0.960 (0.942-0.974) | 0.819 (0.792-0.845) | 0.737 (0.639-0.821) | 0.830 (0.801-0.856) | 9.069 (5.967-13.783) | 0.662 (0.595-0.736) |
| Arm: Artery SUVmax / Blood SUVmax | 163 patients/846 segments | 0.713 (0.669-0.757) | 0.236 (0.208-0.267) | 1.319 | 0.400 (0.332-0.471) | 0.967 (0.951-0.980) | 0.833 (0.806-0.858) | 0.792 (0.700-0.866) | 0.839 (0.811-0.865) | 12.305 (7.817-19.368) | 0.620 (0.553-0.695) |
| Arm: Artery SUVmax / Blood SUVmean | 163 patients/846 segments | 0.705 (0.660-0.75) | 0.236 (0.208-0.267) | 1.548 | 0.455 (0.385-0.527) | 0.913 (0.889-0.934) | 0.805 (0.777-0.831) | 0.619 (0.535-0.698) | 0.844 (0.815-0.87) | 5.249 (3.917-7.033) | 0.597 (0.525-0.679) |
| Arm: Artery SUVmean / Blood SUVmean | 163 patients/846 segments | 0.699 (0.654-0.744) | 0.236 (0.208-0.267) | 1.026 | 0.460 (0.389-0.532) | 0.870 (0.842-0.895) | 0.773 (0.743-0.801) | 0.523 (0.446-0.598) | 0.839 (0.809-0.866) | 3.538 (2.756-4.541) | 0.621 (0.544-0.708) |
| Arm: Artery SUVpeak / Blood SUVpeak | 163 patients/846 segments | 0.717 (0.673-0.76) | 0.236 (0.208-0.267) | 1.075 | 0.420 (0.351-0.492) | 0.947 (0.927-0.963) | 0.823 (0.795-0.848) | 0.712 (0.621-0.792) | 0.841 (0.812-0.867) | 7.980 (5.537-11.501) | 0.612 (0.543-0.69) |
| **Neck-Arm-Thorax: Artery SUVmax / Liver SUVmax** | 163 patients/2185 segments | 0.673 (0.643-0.702) | 0.241 (0.223-0.26) | 0.808 | 0.484 (0.440-0.527) | 0.834 (0.815-0.851) | 0.749 (0.730-0.767) | 0.480 (0.437-0.524) | 0.836 (0.817-0.853) | 2.907 (2.529-3.341) | 0.619 (0.569-0.674) |
| Neck-Arm-Thorax: Artery SUVmax / Liver SUVmean | 163 patients/2185 segments | 0.700 (0.672-0.728) | 0.241 (0.223-0.26) | 1.085 | 0.516 (0.473-0.56) | 0.818 (0.799-0.837) | 0.746 (0.727-0.764) | 0.475 (0.433-0.516) | 0.842 (0.823-0.859) | 2.843 (2.493-3.242) | 0.591 (0.540-0.648) |
| Neck-Arm-Thorax: Artery SUVmean / Liver SUVmean | 163 patients/2185 segments | 0.676 (0.648-0.705) | 0.241 (0.223-0.26) | 0.867 | 0.421 (0.379-0.465) | 0.880 (0.863-0.895) | 0.769 (0.751-0.787) | 0.527 (0.478-0.576) | 0.827 (0.809-0.844) | 3.510 (2.978-4.137) | 0.658 (0.610-0.709) |
| Neck-Arm-Thorax: Artery SUVpeak / Liver SUVpeak | 163 patients/2185 segments | 0.685 (0.656-0.714) | 0.241 (0.223-0.26) | 0.823 | 0.463 (0.420-0.507) | 0.869 (0.852-0.885) | 0.771 (0.753-0.789) | 0.529 (0.483-0.576) | 0.836 (0.818-0.853) | 3.538 (3.031-4.128) | 0.618 (0.570-0.670) |
| Neck-Arm-Thorax: Artery SUVmax / Blood SUVmax | 163 patients/2185 segments | 0.716 (0.689-0.744) | 0.241 (0.223-0.26) | 1.173 | 0.518 (0.474-0.561) | 0.844 (0.826-0.862) | 0.766 (0.747-0.783) | 0.514 (0.471-0.557) | 0.846 (0.828-0.863) | 3.329 (2.897-3.826) | 0.571 (0.521-0.625) |
| Neck-Arm-Thorax: Artery SUVmax / Blood SUVmean | 163 patients/2185 segments | 0.708 (0.68-0.736) | 0.241 (0.223-0.26) | 1.456 | 0.581 (0.537-0.623) | 0.776 (0.755-0.796) | 0.729 (0.709-0.747) | 0.451 (0.413-0.49) | 0.853 (0.834-0.871) | 2.588 (2.306-2.904) | 0.541 (0.487-0.600) |
| Neck-Arm-Thorax: Artery SUVmean / Blood SUVmean | 163 patients/2185 segments | 0.686 (0.657-0.714) | 0.241 (0.223-0.26) | 1.176 | 0.469 (0.425-0.512) | 0.841 (0.823-0.859) | 0.751 (0.733-0.769) | 0.484 (0.440-0.529) | 0.833 (0.814-0.85) | 2.955 (2.560-3.410) | 0.631 (0.581-0.686) |
| Neck-Arm-Thorax: Artery SUVpeak / Blood SUVpeak | 163 patients/2185 segments | 0.709 (0.681-0.737) | 0.241 (0.223-0.26) | 1.07 | 0.529 (0.486-0.573) | 0.831 (0.812-0.849) | 0.758 (0.74-0.776) | 0.499 (0.457-0.541) | 0.847 (0.829-0.865) | 3.135 (2.742-3.583) | 0.566 (0.516-0.621) |

**Supplementary Table 7: Cut-off determination for different SUV-ratios derived from ROC from different artery subgroups in all 163 patients (head 138 patients) with the clinical diagnosis as reference:** Values in parentheses are 95%-confidence intervals. LR, likelihood ratio; NPV, negative predictive value; n.a., not applicable; PPV, positive predictive value; SUV, standardized uptake value.

| **Uptake time** | **Criterion** | **Sensitivity** | **Specificity** | **Accuracy** | **PPV** | **NPV** | **LR+** | **LR-** |
| --- | --- | --- | --- | --- | --- | --- | --- | --- |
| 60 min, N=53, GCA N=7 | Visual, >=1 artery grade 3 | 0.429 (0.099-0.816) | 0.956 (0.849-0.995) | 0.885 (0.766-0.956) | 0.600 (0.147-0.947) | 0.915 (0.796-0.976) | 9.643 (1.943-47.867) | 0.598 (0.314-1.139) |
| 60 min, N=53, GCA N=7 | Visual, >=1 artery grade 2 or 3 | 0.714 (0.290-0.963) | 0.844 (0.705-0.935) | 0.827 (0.697-0.918) | 0.417 (0.152-0.723) | 0.950 (0.831-0.994) | 4.592 (2.010-10.493) | 0.338 (0.104-1.099) |
| 90 min, N=53, GCA N=19 | Visual, >=1 artery grade 3 | 0.684 (0.434-0.874) | 0.970 (0.842-0.999) | 0.865 (0.742-0.944) | 0.929 (0.661-0.998) | 0.842 (0.687-0.940) | 22.579 (3.199-159.347) | 0.326 (0.168-0.633) |
| 90 min, N=53, GCA N=19 | Visual, >=1 artery grade 2 or 3 | 0.789 (0.544-0.939) | 0.848 (0.681-0.949) | 0.827 (0.697-0.918) | 0.750 (0.509-0.913) | 0.875 (0.710-0.965) | 5.211 (2.249-12.071) | 0.248 (0.103-0.600) |
| 120 min, N=57, GCA N=11 | Visual, >=1 artery grade 3 | 0.636 (0.308-0.891) | 0.957 (0.852-0.995) | 0.895 (0.785-0.960) | 0.778 (0.400-0.972) | 0.917 (0.800-0.977) | 14.636 (3.513-60.987) | 0.380 (0.174-0.833) |
| 120 min, N=57, GCA N=11 | Visual, >=1 artery grade 2 or 3 | 0.909 (0.587-0.998) | 0.804 (0.661-0.906) | 0.825 (0.701-0.913) | 0.526 (0.289-0.756) | 0.974 (0.862-0.999) | 4.646 (2.512-8.594) | 0.113 (0.017-0.736) |

**Supplementary Table 8**. **Diagnostic accuracy of visual [18F]FDG PET/CT interpretation stratified by uptake time.** Values in parentheses are 95%-confidence intervals. GCA, giant cell arteritis; LR, likelihood ratio; NPV, negative predictive value; PPV, positive predictive value.

| **Uptake time** | **N** | **Prevalence** | **Sensitivity** | **Specificity** | **Accuracy** | **PPV** | **NPV** | **Positive LR** | **Negative LR** |
| --- | --- | --- | --- | --- | --- | --- | --- | --- | --- |
| 60 min | 53 | 0.135 (0.056-0.258) | 1 (0.590-1) | 0.200 (0.096-0.346) | 0.308 (0.187-0.451) | 0.163 (0.068-0.307) | 1 (0.664-1) | 1.250 (1.080-1.447) | 0.135 (0.056-0.258) |
| 90 min | 53 | 0.365 (0.236-0.510) | 0.895 (0.669-0.987) | 0.212 (0.090-0.389) | 0.462 (0.322-0.605) | 0.395 (0.250-0.556) | 0.778 (0.400-0.972) | 1.136 (0.898-1.436) | 0.496 (0.114-2.151) |
| 120 min | 57 | 0.193 (0.100-0.319) | 1 (0.715-1) | 0.087 (0.024-0.208) | 0.263 (0.155-0.397) | 0.208 (0.108-0.341) | 1 (0.398-1) | 1.095 (1.002-1.197) | 0 (0- n.a.) |

**Supplementary Table 9**. **Diagnostic accuracy of previously published SUVmax/SUVmean(liver) cut-offs stratified by uptake time.** Measures of diagnostic accuracy were calculated using the previously published SUVmax/SUVmean(liver) ratio cut-offs compared with the reference diagnosis. Only the analysis irrespective of scanner type, but with scanner-specific thresholds, is shown. Cut-off >1.3 was used for thorax-abdomen-pelvis arteries for all scanner types. For head-neck-arm arteries, cut-off >1.0 was used for Biograph and Vision scanners, and cut-off >0.8 for Vision Quadra. Values in parentheses are 95%-confidence intervals. GCA, giant cell arteritis; LR, likelihood ratio; NPV, negative predictive value; n.a., not applicable; PPV, positive predictive value; SUV, standardized uptake value.

**A. Uptake time 60 min, N=53, GCA N=7**

| **Parameter** | **Youden threshold** | **Sensitivity** | **Specificity** | **Accuracy** |
| --- | --- | --- | --- | --- |
| Artery SUVmax | 2.525 | 1.000 (0.590-1.000) | 0.467 (0.317-0.621) | 0.538 (0.395-0.678) |
| Artery SUVmean | 1.975 | 0.857 (0.421-0.996) | 0.422 (0.277-0.578) | 0.481 (0.340-0.624) |
| Artery SUVpeak | 1.825 | 1.000 (0.590-1.000) | 0.178 (0.080-0.321) | 0.288 (0.171-0.431) |
| Artery SUVmax/liver SUVmax | 0.818 | 1.000 (0.590-1.000) | 0.378 (0.238-0.535) | 0.462 (0.322-0.605) |
| Artery SUVmax/liver SUVmean | 0.981 | 1.000 (0.590-1.000) | 0.178 (0.080-0.321) | 0.288 (0.171-0.431) |
| Artery SUVmean/liver SUVmean | 0.713 | 1.000 (0.590-1.000) | 0.022 (0.001-0.118) | 0.154 (0.069-0.281) |
| Artery SUVpeak/liver SUVpeak | 0.710 | 1.000 (0.590-1.000) | 0.133 (0.051-0.268) | 0.250 (0.140-0.389) |
| Artery SUVmax/blood SUVmax | 1.052 | 1.000 (0.590-1.000) | 0.333 (0.200-0.490) | 0.423 (0.287-0.568) |
| Artery SUVmax/blood SUVmean | 1.398 | 1.000 (0.590-1.000) | 0.400 (0.257-0.557) | 0.481 (0.340-0.624) |
| Artery SUVmean/blood SUVmean | 0.982 | 1.000 (0.590-1.000) | 0.178 (0.080-0.321) | 0.288 (0.171-0.431) |
| Artery SUVpeak/blood SUVpeak | 1.023 | 1.000 (0.590-1.000) | 0.289 (0.164-0.443) | 0.385 (0.253-0.530) |

**B. Uptake time 90 min, N=53, GCA N=19**

| **Parameter** | **Youden threshold** | **Sensitivity** | **Specificity** | **Accuracy** |
| --- | --- | --- | --- | --- |
| Artery SUVmax | 2.765 | 0.789 (0.544-0.939) | 0.545 (0.364-0.719) | 0.635 (0.490-0.764) |
| Artery SUVmean | 1.935 | 0.842 (0.604-0.966) | 0.394 (0.229-0.579) | 0.558 (0.413-0.695) |
| Artery SUVpeak | 2.095 | 0.789 (0.544-0.939) | 0.455 (0.281-0.636) | 0.577 (0.432-0.713) |
| Artery SUVmax/liver SUVmax | 0.832 | 0.842 (0.604-0.966) | 0.545 (0.364-0.719) | 0.654 (0.509-0.780) |
| Artery SUVmax/liver SUVmean | 1.077 | 0.947 (0.740-0.999) | 0.333 (0.180-0.518) | 0.558 (0.413-0.695) |
| Artery SUVmean/liver SUVmean | 0.867 | 0.895 (0.669-0.987) | 0.455 (0.281-0.636) | 0.615 (0.470-0.747) |
| Artery SUVpeak/liver SUVpeak | 0.895 | 0.737 (0.488-0.909) | 0.636 (0.451-0.796) | 0.673 (0.529-0.797) |
| Artery SUVmax/blood SUVmax | 1.338 | 0.842 (0.604-0.966) | 0.606 (0.421-0.771) | 0.692 (0.549-0.813) |
| Artery SUVmax/blood SUVmean | 1.551 | 0.947 (0.740-0.999) | 0.455 (0.281-0.636) | 0.635 (0.490-0.764) |
| Artery SUVmean/blood SUVmean | 1.155 | 0.947 (0.740-0.999) | 0.394 (0.229-0.579) | 0.596 (0.451-0.730) |
| Artery SUVpeak/blood SUVpeak | 1.136 | 0.842 (0.604-0.966) | 0.455 (0.281-0.636) | 0.596 (0.451-0.730) |

**C. Uptake time 120 min, N=57, GCA N=11**

| **Parameter** | **Youden threshold** | **Sensitivity** | **Specificity** | **Accuracy** |
| --- | --- | --- | --- | --- |
| Artery SUVmax | 2.615 | 0.909 (0.587-0.998) | 0.457 (0.309-0.610) | 0.544 (0.407-0.676) |
| Artery SUVmean | 2.055 | 0.909 (0.587-0.998) | 0.674 (0.520-0.805) | 0.719 (0.585-0.830) |
| Artery SUVpeak | 2.275 | 0.909 (0.587-0.998) | 0.630 (0.475-0.768) | 0.684 (0.548-0.801) |
| Artery SUVmax/liver SUVmax | 0.980 | 1.000 (0.715-1.000) | 0.609 (0.454-0.749) | 0.684 (0.548-0.801) |
| Artery SUVmax/liver SUVmean | 1.328 | 1.000 (0.715-1.000) | 0.565 (0.411-0.711) | 0.649 (0.511-0.771) |
| Artery SUVmean/liver SUVmean | 0.756 | 1.000 (0.715-1.000) | 0.130 (0.049-0.263) | 0.298 (0.184-0.434) |
| Artery SUVpeak/liver SUVpeak | 0.756 | 1.000 (0.715-1.000) | 0.196 (0.094-0.339) | 0.351 (0.229-0.489) |
| Artery SUVmax/blood SUVmax | 1.256 | 1.000 (0.715-1.000) | 0.457 (0.309-0.610) | 0.561 (0.424-0.693) |
| Artery SUVmax/blood SUVmean | 1.960 | 0.909 (0.587-0.998) | 0.565 (0.411-0.711) | 0.632 (0.493-0.756) |
| Artery SUVmean/blood SUVmean | 1.271 | 0.909 (0.587-0.998) | 0.435 (0.289-0.589) | 0.526 (0.390-0.660) |
| Artery SUVpeak/blood SUVpeak | 1.169 | 0.909 (0.587-0.998) | 0.435 (0.289-0.589) | 0.526 (0.390-0.660) |

**Supplementary Table 10**. **ROC-derived patient-level semiquantitative cut-offs stratified by uptake time.** Cut-offs were derived at patient level using all arterial segments. Values in parentheses are 95%-confidence intervals. GCA, giant cell arteritis; SUV, standardized uptake value.

**Supplementary Figure 1. Patient flow.** [^18^F]FDG-PET/CT, [^18^F]-Fluorodeoxyglucose positron emission tomography/computed tomography; GCA, giant cell arteritis.

**Supplementary Figure 2. ROC for artery SUVmax/SUVmean(liver) for 163 patients with 3922 segments** CI, confidence interval

**Supplementary Figure 3. Bland-Altmann plot for SUVmean for 30 patients with 739 segments**

**Supplementary Figure 4. Bland-Altmann plot for SUVmax for 30 patients with 738 segments**
